# Time-Resolved Human Kinome RNAi Screen Identifies a Network Regulating Mitotic-Events as Early Regulators of Cell Proliferation

**DOI:** 10.1371/journal.pone.0022176

**Published:** 2011-07-13

**Authors:** Jitao David Zhang, Cindy Koerner, Stephanie Bechtel, Christian Bender, Ioanna Keklikoglou, Christian Schmidt, Anja Irsigler, Ute Ernst, Özgür Sahin, Stefan Wiemann, Ulrich Tschulena

**Affiliations:** Division of Molecular Genome Analysis, German Cancer Research Center, Heidelberg, Germany; University of Leuven, Belgium

## Abstract

Analysis of biological processes is frequently performed with the help of phenotypic assays where data is mostly acquired in single end-point analysis. Alternative phenotypic profiling techniques are desired where time-series information is essential to the biological question, for instance to differentiate early and late regulators of cell proliferation in loss-of-function studies. So far there is no study addressing this question despite of high unmet interests, mostly due to the limitation of conventional end-point assaying technologies. We present the first human kinome screen with a real-time cell analysis system (RTCA) to capture dynamic RNAi phenotypes, employing time-resolved monitoring of cell proliferation via electrical impedance. RTCA allowed us to investigate the dynamics of phenotypes of cell proliferation instead of using conventional end-point analysis. By introducing data transformation with first-order derivative, i.e. the cell-index growth rate, we demonstrate this system suitable for high-throughput screenings (HTS). The screen validated previously identified inhibitor genes and, additionally, identified activators of cell proliferation. With the information of time kinetics available, we could establish a network of mitotic-event related genes to be among the first displaying inhibiting effects after RNAi knockdown. The time-resolved screen captured kinetics of cell proliferation caused by RNAi targeting human kinome, serving as a resource for researchers. Our work establishes RTCA technology as a novel robust tool with biological and pharmacological relevance amenable for high-throughput screening.

## Introduction

RNA interference has developed into a powerful technology for high-throughput screening. Numerous studies have uncovered novel functions of genes in biological processes within a number of species and conditions. However, most studies have used single end-point analysis as readout for the characterization of the respective phenotypes [1], [2], [3], [4], [5], [6], [7], [8], [9]. An end-point analysis provides merely a “snapshot” of the respective analyzed phenotype, neglecting its development over time. Moreover, in many studies pre-labeling, fixation or cell destruction is required prior to the assay. Few screens have been performed, mostly using high-content screening microscopy, where cellular phenotypes were detected with time resolution [10], [11], [12], [13], [14]. However, even there the timing of events was mostly not considered. Recently, real-time and label-free monitoring of cell proliferation that is based on electrical impedance real-time cell analysis (RTCA) has become available and is just starting to be employed in phenotypic analyses of perturbed cells [13].

The RTCA system is based on the fact that cell membranes consist of a lipid bilayer having high electrical resistance, and that the adhesion of cells can be steadily detected by the gold micro-electrodes at the bottom of wells with electrical impedance as read-out [15], [16], [17]. The strength of impedance is positively correlated with the number of cells having attached to the electrodes and is recorded as cell index (CI) values (Figure S1a). Among other factors, the impedance mainly refects the attached cell number as well as the quality of the cells' interaction with their substrate [10]. Therefore, this method is suitable for quantifying cell proliferation without the need for tagging or modifying the sampled cells, as shown in other technologies relying on cell-impedance [18].

We have exploited such a system to collect continuous and quantitative information on the changes in the electrical impedance that are imposed by RNAi-induced knockdown of genes. Since human kinases and cell cycle proteins are important for cell proliferation and often employed as drug targets, we carried out a human kinome RNAi screen to test the biological and pharmacological relevance of the RTCA system. In this study, we first established impedance measurement as a novel, robust screening tool to monitor cell proliferation by performing screening quality controls (QC) after proper data transformation. Then we integrated the RTCA system into a high-throughput workfow for siRNA transfections. Subsequently, we utilized a human siRNA library targeting 779 kinases and 80 cell cycle genes to analyze cell proliferation in real-time, and monitored the dynamics of the cellular response to knockdown of the respective genes. The obtained real-time profiles of the phenotypes provide novel, direct insights into the dynamics of the knockdown of the involved genes and proteins as well as their impact on the cell system.

## Results

### Cell impedance reﬂects time-resolved cell proliferation

**Figure 1:**
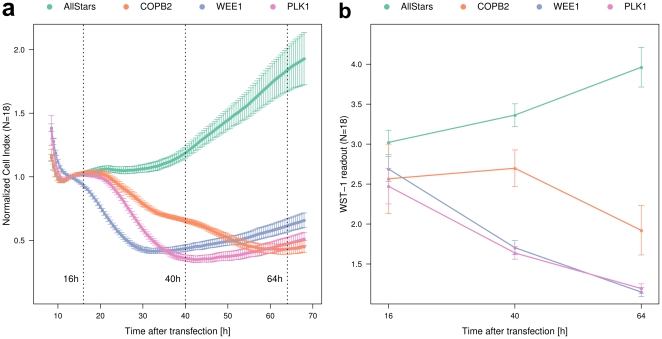
Time kinetics of cell growth after knock-down of indicated control genes and a negative control captured with RTCA system and WST-1 assay. (a) The RTCA system monitors cell growth after transfection of non-targeting control (siAllStars) and cell-growth inhibitors (COPB2, WEE1 and PLK1) from 8 hours up to 68 hours. It records the electrical impedance of growing cells and reports it as cell index. The normalization has been performed according to the manufacturers instructions (Materials and Methods). Three time points at which the WST-1 assay was performed in parallel experiments are indicated in dotted lines. Each curve represents one siRNA knockdown sample, and the error bars indicate the standard deviation of individual cell impedance measurements (*N* 18). (b) WST-1 assay was performed with the same set of siRNA at 16, 40 and 64 hours respectively, supporting the profiles observed with RTCA system (*N* = 18)

With the ability to analyze cell proliferation in a time-resolved manner, we first determined the reproducibility and robustness of the RTCA system. To test three known effectors of cell viability in HeLa cells, PLK1 [19], WEE1 [20], [21] and COPB2 [22], we performed RNAi experiments in 18 biological replicates on six individual microtiter plates. Knockdown of these positive controls for cell proliferation inhibition indeed reproducibly induced a strong reduction of the cell index (Figure 1a) when compared to a non-targeting negative control (siAllStars). The RTCA data was next validated utilizing a WST-1 quantitative colorimetric assay that reﬂects cell viability and proliferation [23]. Very similar phenotypes were observed in the RTCA and WST-1 assays (Figure 1b). The highly reproducible profiles of the positive and negative controls suggest that the onset and dynamics of cellular responses to knockdown of the respective genes are different and specific for the individual targeted genes.

### Data transformation with Cell-Index Growth Rate

**Figure 2:**
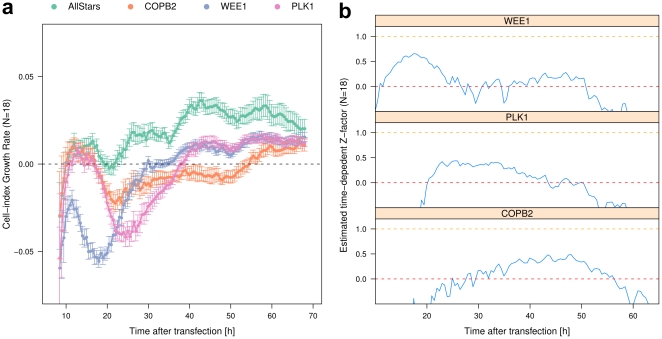
Cell-index growth rate transformation and time-dependent Z-factors of siRNA screen with the RTCA system. (a) The point-wise derivative transformation was applied to the raw data shown in Figure 1 (a), and we name the transformed measure as cell-index growth rate (CIGR). The magnitude of CIGR (its absolute value) measures the transient cell growth rate and its direction (positive, negative and zero) indicates the status of cell growth: in increasing, in recession or constant. Each siRNA sample is represented in one color, with dots indicating mean and error bars indicating standard deviation (*N* = 18). (b) Time-dependent Z-factors calculated with the data of subfigure (a). We have extended the classical Z-factor into time-dependent Z-factor to allow presenting the effect sizes at different time points. The time-dependent Z-factor has been estimated for each positive control of cell growth inhibition. For all the three positive controls, we observed at least one large time window in which the Z-factor is larger than 0, indicating a very robust assay with siRNA. The shifting of these time windows reflects the fact that each positive control has its unique kinetics and distinct time when the maximum effect is reached.

The initial outputs from the commercial software shipped with the RTCA system, the time-series cell index (CI), posed challenges on data analysis that needed to be solved prior to being able to analyze data from high-throughput screens, for instance the reference time point has to be chosen arbitrarily, and the measurement error accumulates at later time points (see Material and Methods). As a solution we introduced the cell-index growth rate (referred to as CIGR later) transformation, by transforming cell index into the point-wise first-order derivative, i.e., the slope of CI curve at each time point (see details in Material and Methods and Figure S2). Analogous to the relationship between velocity and distance in physics, CIGR is the rate of change of cell index and describes the transient status of cell proliferation at any time. The transformation overcomes main pitfalls of using cell index, and we could show that the maximum CIGR in the exponential cell-growth phase is linearly correlated with the cell number (Figure S1b), therefore establishing CIGR as the measure of transient cell proliferation. Figure 2a shows the cell-index growth rate curves by transforming the data presented in Figure 1a. Major differences in the growth characteristics are confined to the first 20–40 hours. The CIGR transformation does not only separate apoptosis inducing siRNAs from the negative control, but also distinguishes different time-kinetics between the cell proliferation inhibitors more clearly. For example, the effect of COPB2 knockdown manifests at later time points (30–60 hours) compared to PLK1 or WEE1, reﬂected by continuous lower CIGR.

### Time-dependent Z-factor establishes RTCA system as a robust tool for high-throughput screening

In order to verify that RTCA is indeed suitable for high-throughput screening, we next estimated time-dependent Z-factors for the data obtained upon knocking down control genes (Material and Methods). The time-dependent Z-factor curves (Figure 2b) indicate that this factor equals or is stably greater than 0 over a large time window (>20 hours) when comparing negative control with each of the three positive control siRNAs. This shows that RTCA indeed allows for the identification of time-resolved significant hits in high-throughput screens. We also observed that the time-windows of maximum effect sizes were shifted among positive controls, indicating distinct kinetics of each gene, and suggesting different time points at which the genes exert their maximum effect on cell proliferation. Together, RTCA allows for the identification of significant hits in real-time high-throughput screens with data transformation.

### Human kinome screen with the RTCA system identifies inhibitors and activators of cell proliferation

Encouraged by the above results, we next performed a whole human kinome screen. In this screen, we knocked down 779 kinases as well as 80 cell cycle genes (referred to as kinases hereafter) in HeLa cells using siRNA libraries. Every knockdown was repeated in two biological replicates with control genes and their biological replicates positioned on each 96-well plate (Table S1).

**Figure 3:**
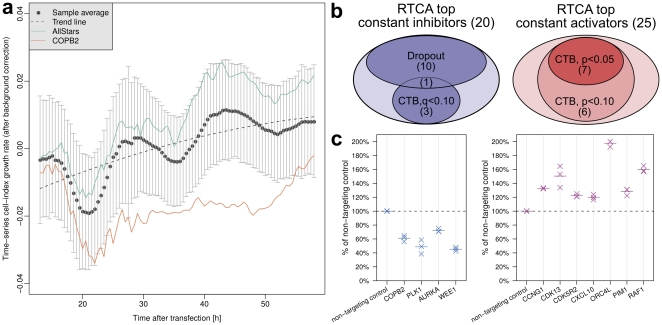
Dynamics of the kinome screen and the overlapping results with conventional end-point assays. (a) The mean cell-index growth rates of all kinase knock-downs (dots) are plotted against time. The error bars (± population standard deviation) indicate the range of CIGR after kinase knock-down, and the dash line by fitting the mean values with a second-order linear model suggests the trend. The dynamics of negative control and one positive control (COPB2) are shown in solid lines for comparison. (b) Overlap of constant hits modulating cell growth identified by the RTCA system and other end-point screens. CTB stands for the cell titer blue viability assay, and Dropout refers to the published drop-out screen with shRNA pools to identify regulators of cell growth by Schlabach et al. [24]. Inhibitors in the CTB screen having marginal significant effects (false-discovery rate q<0.10) and having been identified in RTCA or Dropout are indicated. For activators, uncorrected p-values were employed as otherwise no activating hits would have been obtained. In both activator and inhibitors over 50% of the hits were overlapping between the RTCA system and the conventional end-point screens. (c) Inhibitor (left panel, false-discovery rate of q<0.10) and activator (right panel, one-sided t-test p<0.05) hits of the CTB screen (see Figure S5 for results with one-sided t-test p<0.10). The values are shown in the percentage of non-targeting negative controls without transformation, each in three replicates (crosses). The mean values are shown with short horizontal bars.

In order to test for the presence of global trends in the phenotypes of kinome knockdown, we visualized the time-series CIGR for all tested genes. This generated a population average over all knockdown effects as well as standard deviation bars describing the range of the effects for each time point (Figure 3a). The trend line that was fitted to the data indicated that perturbed cells continue growing, after having passed a lag phase that lasted for about 30 hours post transfection. However, the majority of kinases displayed weak inhibiting phenotypes as compared to the negative control. In contrast, the COPB2 positive control was indeed a strong inhibitor of cell proliferation as the CIGR did not pass the zero line. Interestingly, both negative controls, as well as the population average showed oscillation with a period of approximately 17 hours. This had a strong amplitude at the beginning and became weaker later on. This cycling might reflect the cell cycle synchronization induced by the transfection procedure.

To allow for comparison of different siRNAs across plates, as well as to compare the results of RTCA measurements with that from conventional end-point analysis, we calculated the average CIGR and normalized the data with the z-score method. Normalization proved to efficiently overcome unspecific effects that had not originated from target gene knock-down (Figure S3). This conclusion is supported by the robustness analysis where the separation of controls was monitored across the plates in the screening order (Figure S4). The biological replicates of negative and positive controls in most plates were well distinguished by their z-score, indicative of the assay being robust.

The derived z-scores of average CIGR were used to identify constant modulators of cell proliferation. Top hits from the screen were identified by ranking the genes according to the absolute values of the z-scores and selecting the upper 5% of the genes (i.e. all |z|  = 1.96 equivalently p < 0.05, 45 genes in total). These were clustered into inhibitors and activators of average cell proliferation, respectively (Tables S2 and S3). Time-resolved data on CI and CIGR for each siRNA are provided in Tables S1 and S4, respectively. The average CIGR as well as the z-scores obtained for all siRNAs tested in the screen are provided in Table S5. To test the overlapping of hits with end-point analysis we determined to apply an independent cell titer blue (CTB) cell viability screen (Tables S6 and S7). We initially compared the list of siRNAs and their respective target genes that had inhibited cell proliferation in the RTCA screen with the hits from CTB analysis. Of 20 inhibitors identified by RTCA (|z|  = 1.96), four showed a marginally significant inhibiting effect in the CTB screen (false-discovery rate q < 0.10, Figure 3b), while the other RTCA-hits did not.

Furthermore, we took advantage of an independent data set to test the relevance of our hits. Schlabach et al. [24] had used pooled shRNAs for knockdown of 2,924 genes and tested phenotypic effects in four cell lines using half-hairpin barcodes and microarray deconvolution. Out of the 20 significant inhibitors in the RTCA screen, 11 (55%) showed inhibition in at least one of the cell lines tested by Schlabach et al. (Figure 3b). Another four (20%) of the 20 genes identified by us had not been tested by Schlabach et al. and the remaining five hits were not significant in the shRNA dropout screen. This suggests that RTCA is a highly potent and sensitive screening-tool, and demonstrates the ability to efficiently pick up inhibitors of cell proliferation.

Next, we sought to evaluate the potential activators of cell proliferation that had been identified in the RTCA screen. Again, we compared the RTCA results to data obtained in the CTB-screen as well as to the shRNA dropout screen [24]. Out of 25 siRNAs activating cell proliferation in the RTCA screen, seven (28%) showed an activating effect also in the CTB screen (p < 0.05, Figure 3c), and six more (24%) showed marginal activation effects (p < 0.10, Figure S5). No activators of cell proliferation had been reported from the shRNA dropout screen.

We next wished to investigate whether genes we found to inhibit or activate cell proliferation would be enriched in functional categories. To address this question we performed enrichment analysis with Gene Ontology (GO) biological process (BP) terms and indeed found the inhibitors enriched of GO-terms associated with M-phase and mitosis while the activators of cell proliferation were enriched for GO-terms such as locomotion and positive regulation of migration (Table 1). While these GO-terms and their association to the respective phenotypes are well in line with the biology, the adjusted p-values are (especially in the activator case) only indicative of marginal significance likely because of the small number of genes in the respective categories.

**Table 1:** Function enrichment analysis of both inhibitor and activator hits of cell growth identified during the kinome screening with the RTCA system.

Type	GO ID	GO Biological Process Term	Count/Expected/Size	BH-adj. *p* value
Inhibitor	GO:0000279	M phase	7/0.8/50	3.9×10^−4^
	GO:0007067	Mitosis	6/0.7/50	4.9×10^−4^
	GO:0000280	Nuclear division	6/0.7/43	4.9×10^−4^
	GO:0051301	Cell division	4/0.9/55	3.0×10^−2^
Activator	GO:0007626	Locomotory behavior	4/0.5/18	3.6×10^−2^
	GO:0006935	Chemotaxis	3/0.4/14	3.8×10^−2^
	GO:0051090	Regulation of transcription factor activity	3/0.5/18	3.8×10^−2^
	GO:0030335	Positive regulation of migration	3/0.7/21	4.0×10^−2^
	GO:0007267	Cell-cell signaling	5/1.3/45	4.0×10^−2^

See the Method section for the description of the testing procedure. Identifiers (ID) and descriptions (Term) of over-represented biological process function groups defined by the Gene Ontology (GO) are listed. The column Count/Expected/Size shows (1) the actual count of genes labeled with the GO term/(2) expected count under the null hypothesis of the hypergeometric model/(3) number of genes labeled with the term in all the screened genes. Benjamini-Hochberg multiple-testing correction was applied to the p-values returned by the topGO software and the adjusted p-values are reported.

In all, the cross-validation with two independent end-point datasets and the functional enrichment suggest that RTCA is a powerful tool that is able to identify inhibitors as well as activators of cell proliferation with high sensitivity.

### Analysis of dynamic phenotypes identified a network of mitotic entry and exit genes as early regulators of cell proliferation

**Figure 4:**
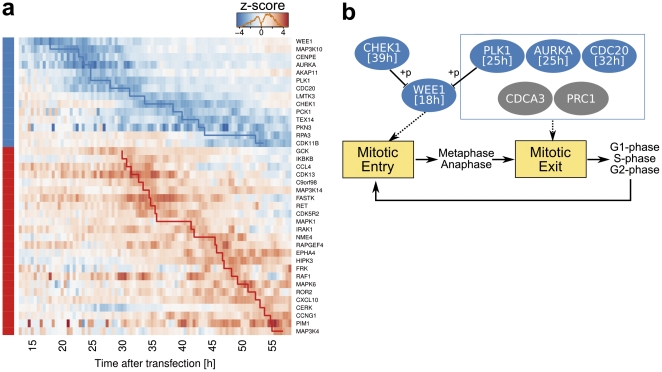
Transient cell-growth modulators identified by the RTCA screen and the interaction network discovered among hits. (a) The time-heatmap showing the kinetics of the significant transient cell-growth modulators identified during the screen (|z|>1.96, minimum time-interval over significance level set to 3 hours), clustered into inhibitors (blue on the left side) and activators (red). The genes in each cluster are ordered increasingly by the time at which the knockdown reaches its maximum effect on the cell-index growth rate (peak-time), indicated by the step curve in blue or red. The color in each cell represents the time-series z-score. (b) The network regulating cellular mitosis event discovered by the screen. Interactions among five inhibitory proteins in subfigure (a) have been reported before and are labeled in blue, the respective peak-time is indicated in brackets. Grey nodes indicate the genes which are not present in the screened library. All three genes that were present and reported to be proteolyzed by the APC-Cdh1 complex and therefore crucial for mitotic exit (in the blue box) were successfully identified. The edges with stunt endings indicate inhibition, and “+p” along the edge indicates effects by phosphorylation. Dotted arrows indicate the involvement of gene products in the cell cycle events.

Having identified inhibitors and activators of cell proliferation, we next asked whether the timing of individual phenotypes could be related to the respective functions of targeted genes. To address this question we first clustered the hits depending on inhibiting and activating effects and according to the time-point where the maximal effect, i.e. the maximum z-score, had been observed (Figure S6). Figure 4a shows inhibitors as well as activators of cell proliferation (p < 0.05, the minimum time-window of significance was 3 hours) ordered by the time-points of their respective maximum effects. While inhibiting phenotypes were observed for several genes already shortly after transfection, the first activating effects took longer to develop. The delay of activators reaching maximum effects compared to inhibitors is robust against algorithms parameter selection (Figure S7).

An early onset of some RNAi phenotypes (15 to 30 hours after transfection) was only seen in the inhibitor set of target genes and this let us then investigate whether the respective genes would be associated with particular gene functions. Interestingly, GO-term analysis revealed that the genes displaying early inhibitory phenotypic effects in the assay, i.e. WEE1, MAP3K10, CENPE, AURKA/STK6, AKAP11 and PLK1, were enriched for GO-terms nuclear division (p<3.9×10^−3^, hypergeometric test, Benjamini-Hochberg adjustment for multiple testing), mitosis (p<3.9×10^−2^), M phase of mitotic cell cycle (p<3.9×10^−2^), and mitotic cell cycle (p<4.9×10^−2^). In contrast to early-onset inhibitors, genes displaying maximum effects at intermediate and late time points (Figure S8a) were not enriched in particular GO-terms. This could indicate that the early-onset phenotypes reflect more direct effects (interruption of cell-cycle mechanisms) while phenotypic changes are increasingly affected by secondary effects at later time points.

To elucidate the biological functions and effects transient modulators have on cell proliferation, data mining tools including STRING [25], Reactome [26], [27] as well as R packages KEGGgraph [28] and RpsiXML [29] were used to query the functional relationships between the hits. By mapping biochemical protein-protein interactions among transient hits, we identified a network of eight cell-proliferation modulators which interact directly and regulate cell proliferation in the HeLa cell system (Figure 4b). Most strikingly, a network of three key genes for mitosis exit, which are rapidly proteolyzed upon induction by APC-Cdh1: AURKA/STK6, PLK1 and CDC20 [30], were identified. All three genes were identified as early (AURKA and PLK1) or early-intermediate (CDC20, reaching the maximum at 32h) modulators, suggesting the sensitivity of the cell system to the mitosis event and demonstrating the advantage of the RTCA system to study transient and dynamic effects. The other two genes having been associated with this pathway (PRC1 and CDCA3/TOME-1) were not present in our screening library. Another gene important for mitosis entry, WEE1, was discovered as the earliest modulator, whereas WEE1s negative regulator CHEK1 (by phosphorylation) reached its maximum effect much later (39h). In order to validate these hits, we next deconvoluted the originally employed pools of four siRNAs to target the indicated genes with individual siRNAs. To this end, each of the four siRNAs contained in the pools of the primary screen was transfected in independent experiments. Figure S9 demonstrates that indeed most siRNAs targeting the respective genes induced a similar phenotype as had been observed with the pools. Normalized CI and CIGR values of the deconvolution experiments are provided in Tables S8 and S9. In order to prove efficient knockdown of the siRNA reagents we performed qRT-PCR experiments after transfection of HeLa cells with each of the individual siRNAs or with the pools. Figure S10 shows that almost all of the siRNAs led to greater than 60% mRNA knockdown thus confirming the efficiency of RNAi.

In conclusion, the time-series cell-growth information gained from RTCA demonstrates its power by discovering key genes involved in cell mitotic entry and exit events as early regulators of cell proliferation.

## Discussion

Here we have described a time-resolved RNAi screen, testing dynamic phenotypic effects that human kinases and cell-cycle genes have on cell proliferation. By applying adequate transformation, we could show with time-dependent Z-factors that the cell-index growth rate (CIGR) is reproducible and renders the technology applicable for screening purposes. Consequently we tested a human kinome siRNA library for dynamic effects of knockdown on cell proliferation. While knockdown of the majority of kinases indeed had slight inhibiting effects on cell proliferation, the cells appeared to be able to overcome these effects and continue growing once a lag phase variable in length has passed. This let us conclude that HeLa cells are indeed sensitive to the loss of these kinases, however, they are fit to overcome the initial effects of the perturbations and they thus are robust from a systems point of view. In addition, an oscillation with a period of 17 hours of population could be observed with high amplitude at the beginning, which becomes smaller at later time points. We assume that the oscillation might visualize cell cycle of the cells, as the cells might be partly synchronized by the transfection procedure. This observation is also confirmed by the reduction of amplitude over time as a de-synchronization of cell cycle will occur. Moreover, a cell cycling time of 16∼18 hours has been observed in HeLa before [31], a similar frequency to that observed by us. We thus conclude that RTCA might serve as a powerful tool to analyze cell cycle changes.

Our screen identified genes having inhibiting as well as activating phenotypes, with more than 50% of hits in both categories validated by a cell titer blue assay or a published shRNA dropout screen [24]. The overlap validates the relevance of RTCA for screening purposes. Interestingly, our screen identified 25 genes having activating phenotypes upon knockdown on top of 20 inhibitor phenotypes. While some of these activators were only marginally significant also in the CTB analysis and, hence, would not have been followed up further, the shRNA screen was not able to pick up any genes having activating phenotypes. RTCA thus adds a new dimension in viability analysis as it allows to identify activating phenotypes on cell proliferation in large-scale RNAi screening with high significance.

We next analyzed the timing of inhibition as well as of activation on cell proliferation. To this end, hits were ordered by the time of their respective maximal effects. Strikingly, the first inhibiting effectors became apparent shortly after transfection, whereas activating effects took much longer to develop. The selection of the assay time in an end-point screen thus reflects a compromise that is likely not able to pick up all phenotypes and thus misses a lot of useful information. GO-term analysis of the early onset inhibitors of cell proliferation revealed an enrichment for GO-terms such as cell cycle and mitosis, being consistent with the transient expression of many cell cycle genes only at confined phases of the cell cycle.

Utilizing this time-resolved data we identified transient cell proliferation modulators and discovered a network of genes that interact with each other and affect cell proliferation in our cell system (Figure 4b). It would have been very difficult to pinpoint this network with conventional end-point assays. The dynamics of components in this network fits their biological functions well, since we observed key cell cycle genes act as early inhibitors and upstream signaling pathway genes as intermediate or late activators. Strikingly we could identify and validate all the genes known to be essential for mitosis exit that are represented in the screening library (CDC20, AURKA/STK6 and PLK1) as early or early-intermediate modulators.

Having determined a number of genes that are involved in mitotic exit, this approach could be exploited in the future, e.g. for improving cancer therapies. Specifically the targeting of the mitotic exit is in line with current anti-mitotic development, as this has been suggested to have enhanced therapeutic capacities than, for example, targeting spindle assembly [32]. Therefore, the RTCA system could potentially be used to discover compounds that target mitotic events, since they may display early effects on cell proliferation as the network of genes identified in this study. This, however, would not easily be possible with traditional end-point assays. Furthermore, it allows investigating cell proliferation kinetics with combinational therapy by applying multiple compounds or RNAi reagents, in order to overcome transient inhibition or activation by single component and achieve constant regulation of cell proliferation [33].

Commonly, protein half-lives are determined in order to predict phenotypic changes. Instead RTCA provides information at what time after the initial perturbation the concentration of a targeted protein has reached a critical concentration below which it is not able any more to perform its biological function. We thus render this a biological half-life kind of information and to have superior content over protein half-life measurements. We are aware that both knockdown efficiency as well as the timing of siRNA-induced silencing are additional parameters that need to be considered in the dynamic analysis of phenotypes. The reagents utilized for the candidates shown in Figure 4 and leading to early phenotypic changes had all similar knockdown efficiencies suggesting that for those genes the timing of RNA knockdown and protein degradation might have similarly contributed to the phenotype. For long-lived proteins, like some structural proteins, the onset of phenotypes could, however, be at late time-points even though RNA knockdown was efficient already early after transfection. While high-throughput half-life data reported a positive correlation between half-life and protein size [34], our data did not verify this association (Figure S8b). This may be due to the small set of proteins that we identified in this screen. However, as we have indeed found a correlation with cell cycle associated GO-terms for early-onset inhibitors and with migration terms for activators we conclude that the biological half-life might be more relevant than protein size. A combination of protein and biological half-life information would nonetheless be ideal as the effects observed with impedance measurement do not distinguish between direct and indirect effects. For example, GO-term enrichment was only visible among the early-onset inhibitors, while late-onset inhibitors did not cluster into specific functional categories. There, the effects of events downstream of the targeted gene could become dominant. Such consecutive order of effects could explain also biphasic dynamic profiles as were observed for some proteins. For example, knockdown of COPB2 (Figure 1) has been described to result in a slow accumulation in remnant structures of ER proteins, eventually leading to a break down of protein secretion and to apoptosis [22]. Loss of that protein thus leads to a consecutive order of events that culminate in cell death while the retrograde trafficking of vesicles from the Golgi complex to the ER has not been reported to be involved in apoptotic pathways. Integration of protein half-life and biological half-life data would thus allow for a direct correlation between biochemical properties of targeted proteins and the cellular fate. Until then, time-resolved analysis of RTCA profiles is a powerful tool to identify the timing in end-point assays, where the maximum effect sizes could be studied (Tables S1 and S6).

In this study we aimed to test the capabilities of the RTCA system in large-scale RNAi screening, using a RNAi-library targeting the human kinome and testing for dynamic phenotypic changes. Considering the limitations of any high-throughput screening, we performed, on top of carrying out an independent CTB screen and integrating a literature dataset, deconvolution experiments that successfully verified some early inhibitors (Figures S9 and S10). More such tests might potentially validate even more modulators having scored less significant in our intial screen, as we found indeed most RNAi-reagents to induce milder inhibiting or activating effects as compared to the negative control. We thus publicize the raw data for deeper mining as well as the R/Bioconductor package RTCA to encourage further use of the data and the software described here.

In summary, we have carried out a human kinome RNAi screen, using RTCA with electrical impedance as output. This screen has validated previously identified inhibitor genes as well as activators of cell proliferation. The high-content of data with respect to time-resolution permits to investigate the dynamics of RNAi phenotypes. Thereby, we identified a network of mitosis-related genes to be among the first displaying cellular effects upon siRNA knockdown. Our data on one hand establishes RTCA technology as a novel tool amenable for high-throughput screening, and on the other hand opens new avenues in the dynamic cellular analysis of phenotypes being induced by RNAi and likely also other perturbations.

## Materials and Methods

### RNAi reagents and cell culture

Human siARRAY - Protein Kinase (G-003500-02) and human siARRAY - Cell Cycle (G-003250-02) libraries were obtained from Dharmacon (Lafayette, US). Catalog numbers of the individual siRNA pools are given in Table S6. Deconvolution experiments were performed with four individual siRNA reagents that had been pooled in the original siARRAY libraries. Order numbers and sequences of these individual siRNAs are provided in Table S10. Negative control siRNA reagents were siAllStars (Qiagen, Hilden, Germany), siGENOME Non-Targeting siRNA Pool #1 (Dharmacon, Lafayette, US). All negative control siRNA reagents were tested side by side in RTCA and CTB assays to verify their lack of effects in the HeLa cell line employed in the experiments (Figure S11). HeLa cells (CCL-2, ATCC) were grown in DMEM medium supplemented with 20 mM L-Glutamine and 10x MEM/NEAA (all Fisher Scientific, Schwerte, Germany).

### Automated siRNA-Transfection and RTCA measurements

The background impedance of the real-time cell analysis system (RTCA, xCELLigence Roche, Penzberg, Germany) E-Plates 96 was performed using the standard protocol provided in the software with 100 µL DMEM-medium containing penicillin/streptomycin, L-Glutamin and 10% FCS (Gibco, Darmstadt, Germany). Following trypsination, cell concentration was determined with a CASY-TT CellCounter (Roche, Penzberg, Germany) and 10,000 HeLa cells were seeded in every well with 100 µL additional DMEM-Medium. E-Plates were positioned in a xCELLigence Real-Time Cell Analyzer MP (Roche, Penzberg, Germany) and baseline levels were recorded. 24 hours later, plates were removed from the incubator and transfection was carried out. Transfection of siRNAs (human siARRAY -Protein Kinase and Cell Cycle libraries) was carried out in a 96-well format using a Biomek FXP liquid handling workstation (Beckman Coulter, Fullerton, US). Prior to transfection, DMEM-medium was removed from the E-plates using a 96 well pipetting head of the liquid handler without touching the cells at the surface of the well bottom. Cells were washed once with 150 µL Optimem (Invitrogen, Karlsruhe, Germany) before adding 40 µL Optimem to each well. In parallel, 1.15 µL X-tremeGENE (Roche, Penzberg, Germany) and siRNA were diluted in 20 µL OPTIMEM, then mixed and incubated for 15 minutes before being added to the E-Plates 96 by the liquid handlers 96 well pipetting head, leading to a final volume of 80 µL per well and a final siRNA concentration of 60 nM (both for pools as well as for individual siRNAs). After 5 hours of incubation in the RTCA MP Station, the transfection mix was removed with the liquid handler, and wells were washed with 150 µL of DMEM-medium and then filled with 200 µL of DMEM-medium before returning the plates to the Real-Time Cell Analyzer MP. Cells were then incubated for 90 hours and impedance was measured every 15 minutes for 25 hours. Thereafter impedance measurement was continued in 60 minutes intervals for another 36 hours. A spline model was built to interpolate the time-series cell-index into a uniform time-interval of 30 minutes between records in tandem. Different negative controls (siAllStars, siGENOME Non-Targeting siRNA Pool #1) were tested side-by-side and displayed similar phenotypes (Figure S11).

### Cell Titer Blue assay

HeLa cells were counted and set to a concentration of 1.25×10^5^ cells/mL. Transfection of 50 nM individual siRNAs (human siARRAY Protein Kinase and Cell Cycle libraries) was carried out in a 96-well format using a Biomek FXP liquid handling workstation (Beckman Coulter, Fullerton, US). siRNAs had been complexed with DharmaFECT transfection reagent (20 µL total volume) for 20 minutes at room temperature, and were then mixed with 80 µL cells and incubated for 48 hours. CellTiter-Blue viability assays (Promega, Mannheim, Germany) were carried out according to the supplier's instructions measuring three replicates of every screening plate. Five µL of CellTiter-Blue reagent were dispensed to every well of 96well plates, plates were gently mixed and then incubated for two hours at 37°C and 5% CO_2_. Then, ﬂuorescence intensities were measured at 485 nm (excitation) and 530 nm (emission) using a Mitras LB940 microplate reader (Berthold Technologies, Bad Wildbad, Germany). Raw-data of the three replicates are provided in Table S6. The results of siRNA transfected samples were then compared to that of non-targeting controls after log-transformation with paired t-test. Multiple-testing correction was performed with the Benjamini-Hochberg method [35]) to yield an estimated upper boundary of local false-discovery rates (q-values). Of activating genes, over 300 had q-values very close to 0.10, which we believed to be caused by the relative small sample size and the ranking approach of the multiple-testing correction. To avoid this potential problem, we made the comparison between result sets by using uncorrected p-values, while reporting data (including the statistical test results) of the CTB assay in the Table S7. Different negative controls (siAllStars, siGENOME Non-Targeting siRNA Pool #1) were tested side-by-side and displayed similar phenotypes (Figure S11).

### WST1 cell proliferation Assay

Cell viability was assayed using the WST-1 Cell Proliferation Reagent (Roche, Penzberg, Germany) according to the manufacturer's instructions.

### qRT-PCR

Total RNA of transfected cell lines was extracted using the Invisorb Spin cell RNA mini kit (Invitek GmbH, Berlin, Germany) and reverse transcribed with the RevertAidTM H Minus First Strand cDNA Synthesis kit (Fermentas, St. Leon-Rot, Germany). Ten ng total RNA was used for each qRT-PCR reaction. Quantitative real-time PCR was performed with probes of the Universal Probe Library (Roche, Penzberg, Germany) for target genes CHEK1, WEE1, PLK1, AURKA, and CDC20 (sequences and probe numbers given in Table S11) Transfections with non-targeting siRNAs siAllStars, siGENOME Non-Targeting siRNA Pool #1 were analyzed as negative controls. Quantification of amplification products was performed with a ABI Prism 7900HT Sequence Detection System (Applied Biosystems, Weiterstadt, Germany). Data was analyzed using the Bioconductor ddCt package [36].

### Bioinformatic and statistical data analysis

Hereafter we use following definitions: in the real-time cell analysis (RTCA) system, the time-series cell electrical impedance is recorded and exported as the cell index (CI) vector 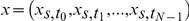 in N time points (N = 91 in the kinome screen, with a measurement time point every 30 minutes from 13h to 58h post transfection) for sample *s*. We denote the set of all screened kinases and cell-cycle related genes (short as kinases hereafter) as 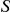, and the complete time domain as 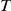. 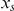 is a unit-less and non-negative 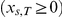 measurement of the electrical impedance. The sample mean and variance between biological replicates of sample *s* at time-point *t* are denoted as 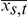 and 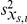
respectively, and the respective standard deviation as 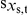. The cell-index growth rate vector is denoted as 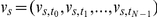, and its derivatives are denoted similarly as the cell-index.

#### Transformation of Cell-Index (CI) into Cell-Index Growth Rate (CIGR)

Two main drawbacks, which prevent employing the CI value directly in a high-throughput screening, are discussed here. Since the CI value is positively correlated to the cell number (Figure S1), and cell numbers vary from well to well on microtiter plates due to the variances of initial seeding and medium change after siRNA transfection, the CI vectors of different samples cannot be compared directly. The manufacturer recommends the normalization as follows: for any given sample set 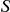, choose an arbitrary time point (denoted as 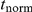) as the normalization time point, and normalize the cell-index for any 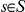 with: 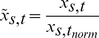
(1)for all 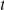. The transformed value 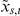 is named as *normalized cell index* by the manufacturer. It has the property that 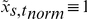. Although this transformation allows to compare the cell growth among samples, it suffers from the arbitrary selection of the 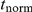. Practically 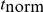 is preferentially chosen at a time point shortly after the siRNA transfection (for instance 10 hours). However, there is no rule defining how it should be selected and we indeed observed effects caused by the arbitrary selection of 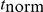.

Besides the arbitrariness of 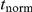, using (normalized) cell-index directly as the measure of cell-growth causes the problem of the error propagation in the time domain 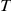. Figure S2(a) and S2(b) show the distributions of 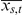 (mean cell-index of kinase biological replicates) and of 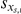 (standard deviation of cell-index of kinase biological replicates) over time for all 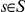. Especially Figure S2(b) suggests that in general the cell-index variance of biological replicates increases along the time. This is most likely to be explained by the error propagation in the time domain: the cell-index measures the cell-growth in an accumulative manner, that is, 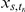 reflects the accumulative growth for 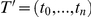 but not the transient cell growth status (Figure S1). Trivial departures from earlier time points will accumulate to large variances in later time points, and causing the increasing 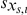 in the time domain 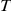.

#### Data transformation with the Cell-Index Growth Rate (CIGR)

To overcome these challenges, we introduced the Cell-Index Growth Rate (CIGR) transformation. We define the cell-index growth rate as: 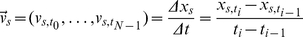
(2)given sample 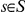 and 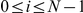. Therefore the CIGR is the rate of change of cell index. Note that by definition the CIGR is a vector physical quantity. The scalar absolute value (magnitude) of 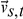 measures the absolute transient cell growth at 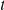, and its sign (direction) determines the status of cell growth: increasing (>0), in recession (<0) and constant ( = 0). Analogous to the relationship between velocity and position in physics, the CIGR measures the transient cell-growth independent of its history, namely all the time points that have been recorded before a new time point is observed, once the cell number has been fixed. In the following discussions we use 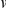 instead of 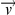 for simplicity.

Applied to the screening data, the CIGR transformation calculates the point-wise first derivative for all 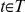 of sample 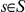 (using diff function of base function in R, see the documentation of RTCA software package for the computational details). Figure S2(c) and S2(d) illustrate the distributions of 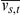
and 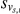 of all screened samples in the same way applied to the untransformed cell-index. Figure S2(c) suggests that the CIGR of all screened samples are evenly distributed in the time domain 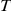, with the positive control (siRNA against COPB2) and the negative control (non-targeting siAllStars) still well separated. We observe that the variances of cell-index growth rate do not steadily increase with the time (Figure S2(d)) but rather remain constant. We observe, intriguingly, a moderate reduction of average sample variances at about 30 hours after the transfection. We assume this could have been caused by the time-lapse to establish stable siRNA knockdowns.

Therefore the CIGR transformation is free of choosing 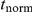 and prevents error propagation in the time domain. Another gain of the transformation is that it is now possible to intuitively compare the transient cell growth of sample 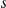 between any two time points 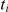 and 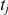,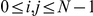, by comparing 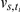 and 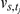 directly.

#### Time-dependent Z-factor

The originally defined Z-factor is a value between 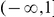 measuring the statistical effect size, judging how well the negative and positive controls can be separated in one assay and consequently whether the assay is suitable for high-throughput screening [37]. To better utilize the time-series information and to capture the statistical effect sizes in the time domain, we have extended the Z-factor to a vector (tuple) of Z-factors indexed by time. The conventional definition of estimated Z-factor according to Zhang et al. [37] was
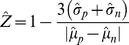
(3)


Four parameters are required to estimate the Z-factor: the means and standard deviations of both the positive (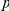) and negative (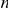) controls. By extending the Z-factor into the time domain 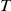, the *time-dependent Z-factor* for 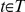
is defined as
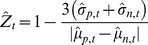
(4)


The time-series Z-factor allows us to capture the statistical effect size of the time-series high-throughput screenings. Since in the RTCA we have used three positive inhibitory controls, we have estimated the time-dependent Z-factor for each of them based on the sample mean and standard deviation of cell-index growth rates 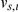 and illustrated them in Figure 2b. Z-scores of replicates were summarized by the square root of the mean squared value of the replicates (root mean square).

#### Average Cell-index Growth Rate

To compare the RTCA screen with conventional end-point assays, we calculate the *average cell-index growth rate* over the time domain 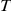 defined by 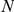 time points, by
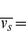
(5)for all 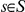. The average cell-index growth rate 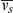 is equivalent to the integral of the cell-growth 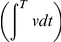, comparable to the results of end-point analysis.

Since we observed effects of experimental condition's change on the average cell-index growth rate between different plates (Figure S3), we performed the *z-score* normalization of 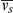 in each 96-well microtiter plate after checking the normality of the sample value distributions [38], [39]. The resulting z-scores based on the average cell-index growth rate, 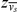, are then used to perform a ranking of the samples. Hits were selected by setting a cut-off at ±1.96, corresponding z-scores deviating about two standard deviations from the mean (equivalent to a significance level of *α* = 0.05 (p<0.05). Figure S4 demonstrates the robustness of the screening by plotting the average cell-index growth rate against the screening plates in the screening time order.

#### Time-series z-score of cell-index growth rate and watershed algorithm

In order to identify transient cell-growth regulators based on the CIGR, we have performed the *time-series z-score normalization* described by the following formula:
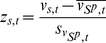
(6)where 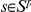, 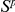 defines the set of all the kinase siRNA samples on one 96-well microtiter plate. The normalities of the distribution of 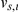 were checked with quantile-quantile-plots and Shapiro-Wilk test. The distributions of resulting time-series z-scores are shown in the Figure S2(e). And the distribution of standard variances of biological replicates based on the time-series z-score is shown in the Figure S2(f).

To identify transient activators/inhibitors, three significance levels (*α* = 0.1, 0.05 and 0.01) and eight minimum time intervals over significance level (1.5h-6h) were plugged in the ﬂooding watershed algorithm (Figures S6 and S7).

#### GO Enrichment Analysis

The enrichment analysis of GO-terms was performed using both the DAVID Bioinformatics Resources 6.7 [40] and the Bioconductor package topGO [41]. Overlapping significant GO terms are reported with the adjusted p-value (Benjamini-Hochberg method [35]) of the results derived from the topGO package.

#### Software availability

All computational and statistical analysis was performed with R-packages of the Bioconductor [42] platform, unless otherwise specified. To make high-throughput data analysis with the RTCA system amenable for public use, we implemented the open-source software package ‘RTCA’ in R on the Bioconductor platform. It imports primary data from the RTCA system and performs data analysis and visualization. The package is freely available at the Bioconductor website [43].

## Supporting Information

Figure S1 The cell impedance (cell-index) is positively correlated with the cell number and the cell-index growth rate reflects the cell-growth rate. (a) Different numbers of HeLa cells (0, 100, 300, 1,000, 3,000, 10,000 and 30,000) were seeded in the xCELLigence system and the cell growth was recorded by measuring the electrical impedance (cell-index). We observe the initial number of cells is indeed correlated with higher cell-indices. In case of too many cells seeded, the cells enter stationary phase following the exponential growth (10,000 and 30,000 cell group) after certain time. (b) With the cell-index growth rate transformation, the data of subfigure (a) is illustrated as the first-degree derivative of the cell-index curve at each time point. It is to note that the maximum cell-index growth rate in the exponential growth is linearly positively correlated with the cell number in a certain range (the three dash lines indicate the CIGR at 3, 1, and 0.33, corresponding to the initial cell number group of 30,000, 10,000 and 3,000). This suggests that the cell-index growth rate reflects the transient cell growth rate.
(PNG)

Figure S2 Cell-index growth rate (CIGR) transformation stabilizes the variance over time, enabling background correction and can be normalized by the z-score method. (a) The distribution of cell-indices in the kinome screening represented by the average value of biological replicates (dots) and the population standard deviation of the average values (error bars indicate ±s.d., also applied hereafter unless otherwise specified) over time. It is obvious that the population standard deviation of the average cell-index increase drastically over the time, and from the cell-index it is not easy to intuitively tell the transient cell growth status at any given time. Green and red curves indicate the average cell-indices of negative control siAllStars and one positive control COPB2. (b) The distribution of sample standard variations of biological replicates over time. Similarly to the average cell-index, the variance between the biological replicates of individual siRNAs are increasing along the time, reflecting the fact that the variances of cell-index measurements accumulates along the time and the errors are propagated. (c) The distribution of transformed cell-index growth rates in the kinome screening, represented by the average value of biological replicates (dots) and the population standard deviation. The transformation stabilizes the variance along the time, and the positive and negative control can still be well distinguished. No plate background correction is performed here compared to the figure 3(a) in the main text. (d) The distribution of standardized variances of biological replicates suggest that the transformation also stabilizes the variances of biological replicates. We note there is a reduction of variances at about 30 hours, and speculate it might be caused by the establishment of the stable siRNA knockdown. (e) The normalization of the cell-index growth rates using the z-score method, showing the average z-score of the kinases over time (dots) and the population standard deviation (error bars), also with the negative and positive controls. (f) The distribution of standardized variances of z-scores of biological replicates over time. The reduction of general variability at around 30 hours is still noticeable but weakend.
(PNG)

Figure S3 The xCELLigence technology is highly sensitive to perturbations and, hence, requires robust normalization to compare data from different experiments. (a) Each dot indicates the average cell-index growth rate (CIGR) of one siRNA knockdown sample over the measurement time. The samples from the same parent plate of the kinome library are depicted in the same color. For each parent plate two biological replicates were performed. The plates are shown in the same order of being screened, and in each 96-well plate the samples are shown in the order from A01 (top-left) to H12 (bottom-right). Thus the figure shows the variances of the un-normalized average cell-index growth rate within and across the screening plates. We observe that the variances within plates are similar, whereas there was an abrupt rise of the average CIGR from the middle of the screening. This was a suspected surprise: our experiment protocol showed that exactly before the screening of the parent plate 7 (orange), the CO_2_ supplier was changed, resulting in an overall increase in measured values. This reflects the sensitivity of the xCELLigence system and calls for correction of plate effects and normalization procedures. (b) Normalization with the z-score method overcame the plate effect and made samples within and across plates comparable. Note that here we have illustrated the process to normalize the average cell-index growth rate as example by reducing the time-series data to one dimension, but the z-score normalization was also applied to the whole time-series data.
(PNG)

Figure S4 Robustness of the xCELLigence screen. Eleven parental 96-well plates with siRNAs targeting 779 kinases as well as 80 cell cycle genes were screened in duplicate. Biological replicates of siRNAs targeting control genes (siCOPB2, siWEE1, siPLK1) as well as a non-targeting control (siAllStars) were present on all plates. Each vertical string (light gray) represents one 96-well plate, and the distribution of the normalized average cell-index growth rates (z-score) is shown with dots: gray dots indicate samples, siAllStars in green, COPB2 in orange, WEE1 in blue and PLK1 in violet. It can be observed that in most screening plates the positive controls can be well separated from the negative controls, and the samples are evenly distributed in the range of normalized data, demonstrating the robustness of the screen.
(PNG)

Figure S5 Marginally significant activators (significance level p<0.10 in one-sided t-test) identified in the CTB screening. Crosses indicate the values of siRNA transfected samples as the percentage of negative controls in three biological replicates, and the short horizontal bars indicate the mean value of the replicates.
(PNG)

Figure S6 The principle of the flooding watershed algorithm and the number of hits on different parameter selections. (a) For the sake of simplicity, we only discuss selecting the positive regulators, the sample principle however also applies to negative hits. To select transient cell-growth modulators, one puts water sources in each regional minimum (blue regions) of the curves in positive, and flood the relief from the sources. To determine whether a cell-index growth rate curve has a transient significant region, two parameters have to be defined: the significance level (analogously the water level) and the minimum time window in which the CIGR is *always* over the significance level, both shown in the figure. Using a time-window instead of a single time point helps to eliminate too transient hits (for example those ones that are significant at only one of the 91 measurement points). It is obvious that the higher the significance level is, or the wider the time window is, the less hits will be identified. (b) The number of identified cell growth regulators (both activating and inhibiting) when adjusting the two parameters of the algorithm introduced in (a). The time-window has been set from 1.5 hours (3 measurement points) to 6 hours (12 measurement points), and the p value cut-off has been set to 0.01, 0.05 and 0.10 (corresponding to the z-score cut-off of |z|>1.64, |z|>1.96 and |z|>2.33 respectively). As expected, the number of hits reduce as the p-cutoff becomes more strict as well as the minimum time interval over significance becomes longer.
(PNG)

Figure S7 The average time to reach the maximum effect on the cell-index growth rate of inhibitors is shorter than that of activators, independent of the parameter selection. Similar as in the Figure S6, we tested 24 combinations of the significance level (p<0.01, p<0.05 and p<0.10) and the time-window interval (shortened as TW, 1.5 hour to 6 hours), and compared the distribution of the time after transfection at which the siRNA knockdown reaches the maximum effect on the CIGR. In all the cases we observe the average time required by the inhibitors is shorter than the activators (p<0.05 for all the combinations, Student' t-test).
(PNG)

Figure S8 Distribution of time after transfection at which the siRNA knock-down reaches the maximum effect on the cell-index growth rate (‘peak-time’) is linked to the biological function but not the protein size. (a) The distribution of the ‘peak-time’ of all the significant (p<0.05, time-window >3 h) transient modulators of cell growth, which can be divided into three groups: early (<30 hour), intermediate (>30 and <45 hour) and later effectors (>45 hour). The early phase differ from the other two phases in the way that it includes the inhibitory effectors exclusively. (b) The time at which the siRNA knockdown reaching the maximum effect is not significantly linearly correlated with the protein size, neither for the activators or the inhibitors. However, as suggested by the function enrichment analysis and the network analysis of hits discussed in the main text, the peak-time is indeed correlated with the biological function of the genes.
(PNG)

Figure S9 Normalized cell index for five genes identified in the primary RTCA screen as inhibitors of cell proliferation (Figure 4). Pools of four individual siRNAs had been transfected in the primary screen and were deconvoluted to also test the siRNAs (indicated with _1 to _4) individually. AllStars negative control was included in all experiments as negative control. Dotted vertical lines at the 8 hour timepoint indicate the time used for normalization of data.
(PNG)

Figure S10 Efficiency of mRNA knockdown of RTCA inhibitor hits measured by qRT-PCR. Pools of four individual siRNAs had been transfected in the primary screen and were deconvoluted to also test the siRNAs (indicated with _1 to _4) individually. SiAllStars negative control as well as siGenome non-targeting control pool #1 were included in all experiments as negative controls. Data was normalized to the effects induced by AllStars control. Results of three technical replicates are shown and error bars indicate the standard deviation.
(PNG)

Figure S11 Comparison of negative control siRNAs. SiAllStars and siGenome non-targeting control pool #1 siRNAs were transfected into HeLa cells, and induced effects were monitored by RTCA (a) and CTB (b) assays. Results of six biological replicates are shown and error bars indicate the standard deviation. In the CTB assay the indicated numbers of cells were transfected, 10,000 cells were seeded in the RTCA assay.
(PNG)

Table S1 Normalized cell index (CI) obtained after knockdown of 779 kinases (plates 1-10) and 80 cell cycle genes (plate 11) in the RTCA screen. PLK1, WEE1 and COPB2 siRNAs were included as targeting controls, while siAllStars was included as non-targeting control on each plate. The screen was performed in two biological replicates for every siRNA. Raw CI values obtained for every sample at every timepoint were normalized to the CI at 8h after transfection.
(CSV)

Table S2 Kinases and cell cycle genes showing constant cell-growth inhibiting effects in the RTCA screening. Annotation: **Rack**: The parent plate in the kinome (or cell cycle) library, plates 1-10 include kinases, and 11 contains cell cycle related genes. **Well**: The well position (A01-H12) on each 96-well microtitre plate. **CatalogNumber**: Catalog number of the siRNA in the library. **GeneSymbol**: Gene symbol annotation shipped along with the library. **EntrezGeneID**: Entrez GeneID. **Accession**: RefSeq accession number. **Zscore**: z-score of the siRNA knockdown sample. **OfficialGeneSymbol**: Current official HGNC Gene Symbol (May 2011). **Identified in Ref 1. as inhibitor of … cell line(s)**: In which cell line(s) were the respective genes identified as cell-growth inhibitors in a shRNA dropout screen (Schlabach et al, Science 319, 620 (2008)) (see the main text for more details), ‘-’ indicates not significant in any of the cell lines, ‘Not Tested’ indicate the gene was not tested in the screening of reference. **Identified as Inhibitor in the CTB Screening**: Whether the siRNA identified as cell-growth inhibitors in the CTB screening (q<0.10), ‘-’ indicates not significant in the CTB screening.
(XLS)

Table S3 Kinases and cell cycle genes showing constant cell-growth activating effects in the RTCA screening. Annotation: **Rack**: The parent plate in the kinome (or cell cycle) library, plates 1-10 include kinases, and 11 contains cell cycle related genes. **Well**: The well position (A01-H12) on each 96-well microtitre plate. **CatalogNumber**: Catalog number of the siRNA in the library. **GeneSymbol**: Gene symbol annotation shipped along with the library. **EntrezGeneID**: Entrez GeneID. **Accession**: RefSeq accession number. **Zscore**: z-score of the siRNA knockdown sample. **OfficialGeneSymbol**: Current official HGNC Gene Symbol (May 2011). **Identified as activator in the CTB Screening**: Whether the siRNA identified as cell-growth activators in the CTB screening (one-sided t-test with indicated p-values), ‘-’ indicates not significant in the CTB screening.
(XLS)

Table S4 Normalized cell index growth rate (CIGR) obtained after knockdown of 779 kinases (plates 1-10) and 80 cell cycle genes (plate 11) in the RTCA screen. PLK1, WEE1 and COPB2 siRNAs were included as targeting controls, while siAllStars was included as non-targeting control on each plate. The screen was performed in two biological replicates for every siRNA. Raw CI values obtained for every sample at every timepoint were transformed to CIGR values as described in Materials and Methods.
(XLS)

Table S5 Average normalized CIGR and z-scores obtained after knockdown of 779 kinases (plates 1-10) and 80 cell cycle genes (plate 11) in the RTCA screen. PLK1, WEE1 and COPB2 siRNAs were included as targeting controls, while siAllStars was included as non-targeting control on each plate. The screen was performed in two biological replicates for every siRNA. Raw CI values obtained for every sample at every timepoint were transformed to normalized CIGR values and z-scores were calculated for every siRNA as described in Materials and Methods.
(XLS)

Table S6 Raw data obtained after knockdown of 779 kinases (plates 1-10) and 80 cell cycle genes (plate 11) in a CellTiter-Blue viability screen. PLK1, GAPDH genes were included as targeting controls, while siCONTROL TOX, siCONTROL RISC-free and siCONTROL non-Targeting siRNA pool #1 were used as non-targeting controls on all plates. The screen was performed in three biological replicates for every siRNA. Fluorescence intensities were measured at 530 nm and the recorded data for each replicate are given in columns G-I.
(XLS)

Table S7 Results obtained from the CellTiter-Blue viability screen. PLK1, GAPDH genes were included as targeting controls, while siCONTROL TOX, siCONTROL RISC-free and siCONTROL non-Targeting siRNA pool #1 were used as non-targeting controls on all plates. The screen was performed in three biological replicates for every siRNA. Fluorescence intensities were measured at 530 nm and values were transformed to the natural logarithm (ln) for each replicate (given in columns D-F). These values were compared to the corresponding values of the non-targeting control and a two-sided student's t-test was performed. In column G, the values for the t-statistics, in column H the resulting p-values are depicted. The Benjamini-Hochberg method was used to yield an estimated upper boundary of local false-discovery rates (q-values, depicted in column I).
(XLS)

Table S8 Normalized cell index (CI) obtained after knockdown of five hits (depicted in figure 4(b)) obtained from the RTCA screen. HeLa cells were transfected in two biological replicates with the siRNA pools used in the screen as well as with each of the individual siRNAs contained in the pools. SiAllStars and siGenome non-targeting siRNA pool #1 were included as non-targeting controls. Raw CI values obtained for every sample at every timepoint were normalized to the CI at 8h after transfection.
(XLS)

Table S9 Cell index growth rate (CIGR) obtained after knockdown of five hits (depicted in figure 4(b)) obtained from the RTCA screen. HeLa cells were transfected in two biological replicates with the siRNA pools used in the screen as well as with each of the individual siRNAs contained in the pools. SiAllStars and siGenome non-targeting siRNA pool #1 were included as non-targeting controls. Raw CI values obtained for every sample at every timepoint were transformed to CIGR values as described in Materials and Methods.
(XLS)

Table S10 Gene Symbols, order numbers (Dharmacon), and sequences of individual siRNA reagents employed in deconvolution experiments. The same siRNAs were contained in aequimolar concentrations also in the siRNA-pools employed in the original RTCA as well as the CTB screens.
(XLS)

Table S11 Gene symbols, Roche UPL probe numbers and primer sequences employed in qRT-PCR verification of knockdown after transfections with siRNAs targeting the respective genes.
(XLS)
