# Supplementary figures and images for: Time-Resolved Human Kinome RNAi Screen Identifies a Network Regulating Mitotic-Events as Early Regulators of Cell Proliferation
Source: PLoS One. 2011 Jul 13;6(7):e22176. doi: 10.1371/journal.pone.0022176 (PMC3135613; doi:10.1371/journal.pone.0022176)

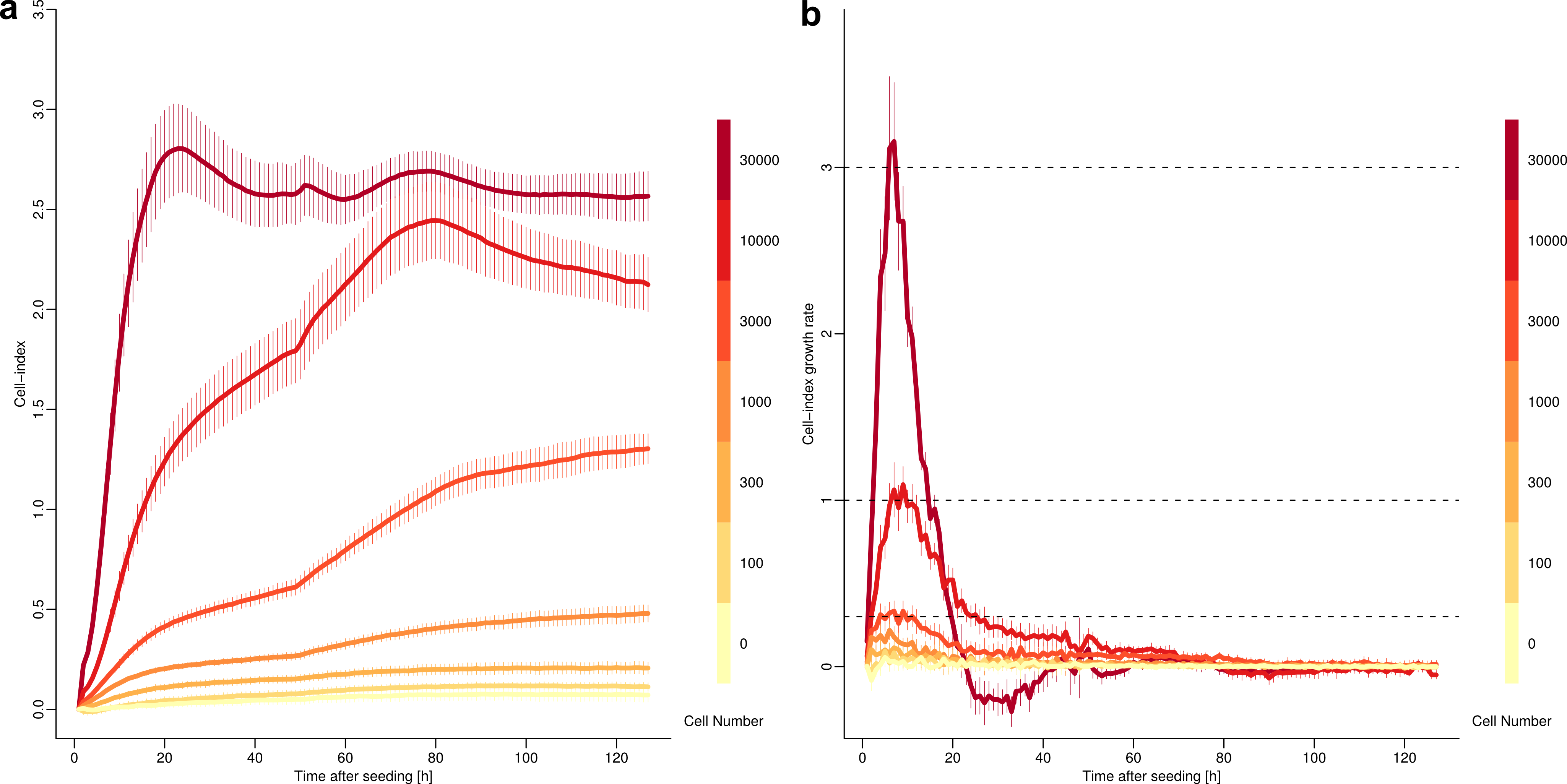

Supplement: Figure S1 — The cell impedance (cell-index) is positively correlated with the cell number and the cell-index growth rate reflects the cell-growth rate. (a) Different numbers of HeLa cells (0, 100, 300, 1,000, 3,000, 10,000 and 30,000) were seeded in the xCELLigence system and the cell growth was recorded by measuring the electrical impedance (cell-index). We observe the initial number of cells is indeed correlated with higher cell-indices. In case of too many cells seeded, the cells enter stationary phase following the exponential growth (10,000 and 30,000 cell group) after certain time. (b) With the cell-index growth rate transformation, the data of subfigure (a) is illustrated as the first-degree derivative of the cell-index curve at each time point. It is to note that the maximum cell-index growth rate in the exponential growth is linearly positively correlated with the cell number in a certain range (the three dash lines indicate the CIGR at 3, 1, and 0.33, corresponding to the initial cell number group of 30,000, 10,000 and 3,000). This suggests that the cell-index growth rate reflects the transient cell growth rate. (PNG) [file pone.0022176.s001.png]

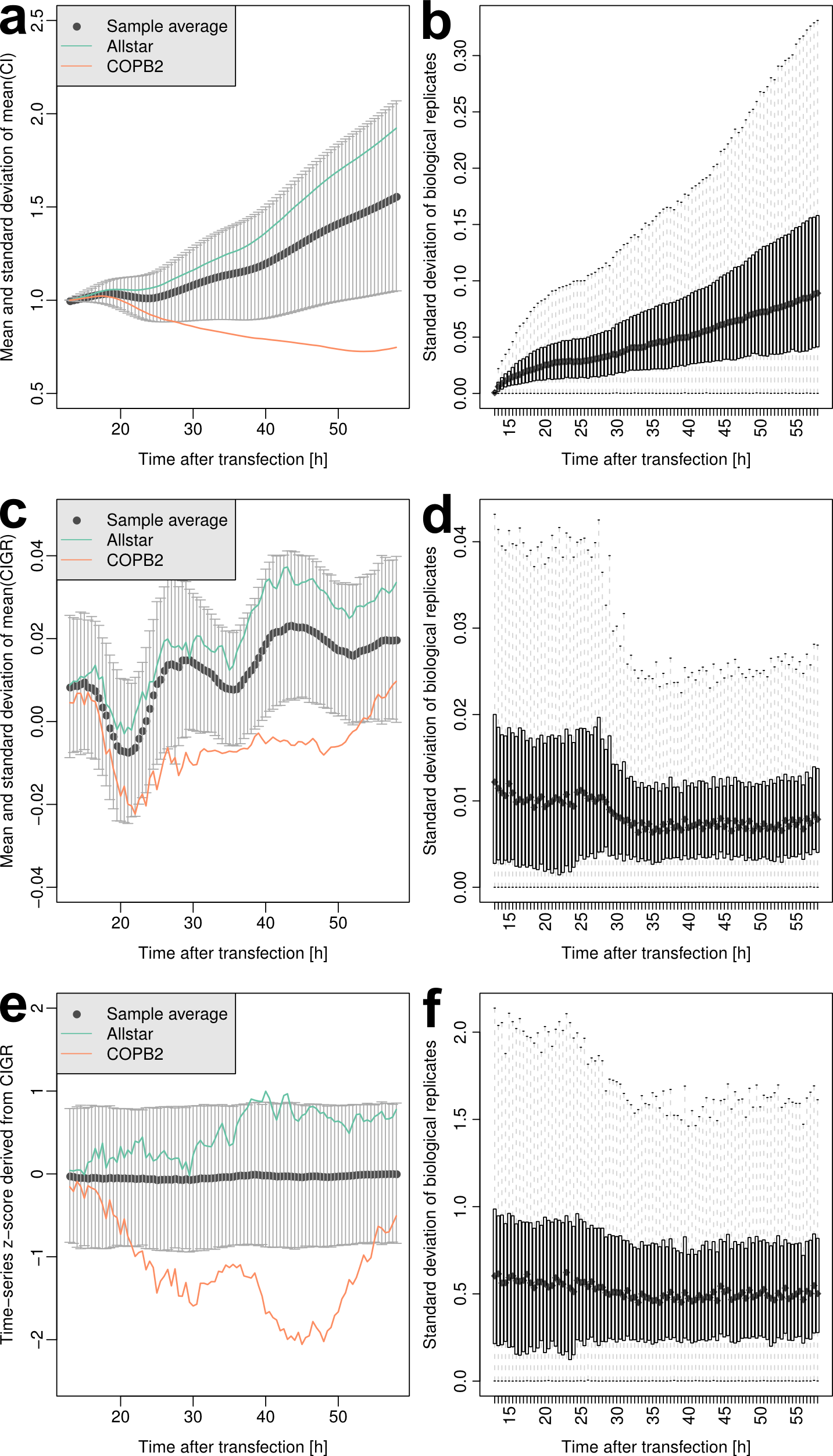

Supplement: Figure S2 — Cell-index growth rate (CIGR) transformation stabilizes the variance over time, enabling background correction and can be normalized by the z-score method. (a) The distribution of cell-indices in the kinome screening represented by the average value of biological replicates (dots) and the population standard deviation of the average values (error bars indicate ±s.d., also applied hereafter unless otherwise specified) over time. It is obvious that the population standard deviation of the average cell-index increase drastically over the time, and from the cell-index it is not easy to intuitively tell the transient cell growth status at any given time. Green and red curves indicate the average cell-indices of negative control siAllStars and one positive control COPB2. (b) The distribution of sample standard variations of biological replicates over time. Similarly to the average cell-index, the variance between the biological replicates of individual siRNAs are increasing along the time, reflecting the fact that the variances of cell-index measurements accumulates along the time and the errors are propagated. (c) The distribution of transformed cell-index growth rates in the kinome screening, represented by the average value of biological replicates (dots) and the population standard deviation. The transformation stabilizes the variance along the time, and the positive and negative control can still be well distinguished. No plate background correction is performed here compared to the figure 3(a) in the main text. (d) The distribution of standardized variances of biological replicates suggest that the transformation also stabilizes the variances of biological replicates. We note there is a reduction of variances at about 30 hours, and speculate it might be caused by the establishment of the stable siRNA knockdown. (e) The normalization of the cell-index growth rates using the z-score method, showing the average z-score of the kinases over time (dots) and the population [file pone.0022176.s002.png]

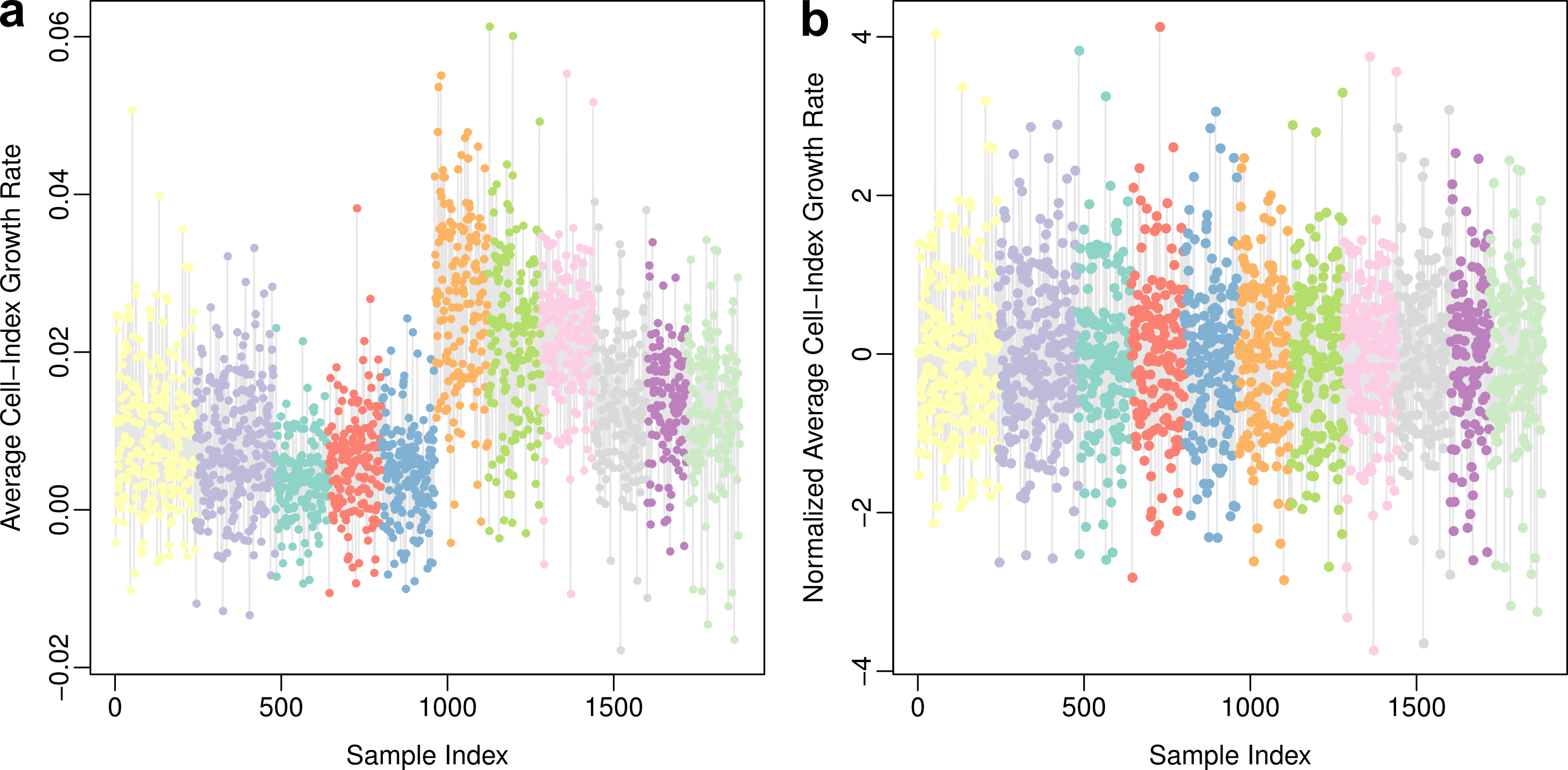

Supplement: Figure S3 — The xCELLigence technology is highly sensitive to perturbations and, hence, requires robust normalization to compare data from different experiments. (a) Each dot indicates the average cell-index growth rate (CIGR) of one siRNA knockdown sample over the measurement time. The samples from the same parent plate of the kinome library are depicted in the same color. For each parent plate two biological replicates were performed. The plates are shown in the same order of being screened, and in each 96-well plate the samples are shown in the order from A01 (top-left) to H12 (bottom-right). Thus the figure shows the variances of the un-normalized average cell-index growth rate within and across the screening plates. We observe that the variances within plates are similar, whereas there was an abrupt rise of the average CIGR from the middle of the screening. This was a suspected surprise: our experiment protocol showed that exactly before the screening of the parent plate 7 (orange), the CO2 supplier was changed, resulting in an overall increase in measured values. This reflects the sensitivity of the xCELLigence system and calls for correction of plate effects and normalization procedures. (b) Normalization with the z-score method overcame the plate effect and made samples within and across plates comparable. Note that here we have illustrated the process to normalize the average cell-index growth rate as example by reducing the time-series data to one dimension, but the z-score normalization was also applied to the whole time-series data. (PNG) [file pone.0022176.s003.png]

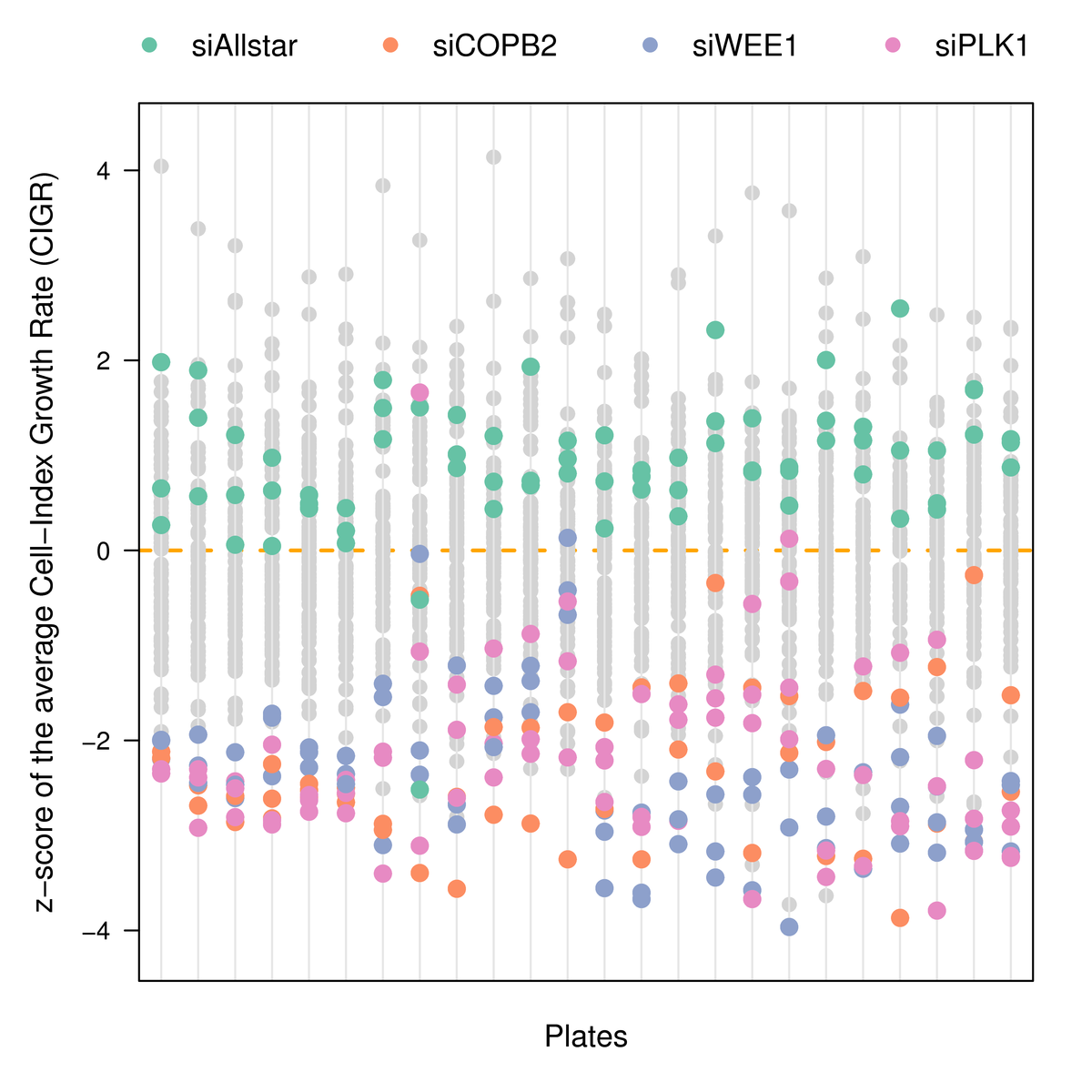

Supplement: Figure S4 — Robustness of the xCELLigence screen. Eleven parental 96-well plates with siRNAs targeting 779 kinases as well as 80 cell cycle genes were screened in duplicate. Biological replicates of siRNAs targeting control genes (siCOPB2, siWEE1, siPLK1) as well as a non-targeting control (siAllStars) were present on all plates. Each vertical string (light gray) represents one 96-well plate, and the distribution of the normalized average cell-index growth rates (z-score) is shown with dots: gray dots indicate samples, siAllStars in green, COPB2 in orange, WEE1 in blue and PLK1 in violet. It can be observed that in most screening plates the positive controls can be well separated from the negative controls, and the samples are evenly distributed in the range of normalized data, demonstrating the robustness of the screen. (PNG) [file pone.0022176.s004.png]

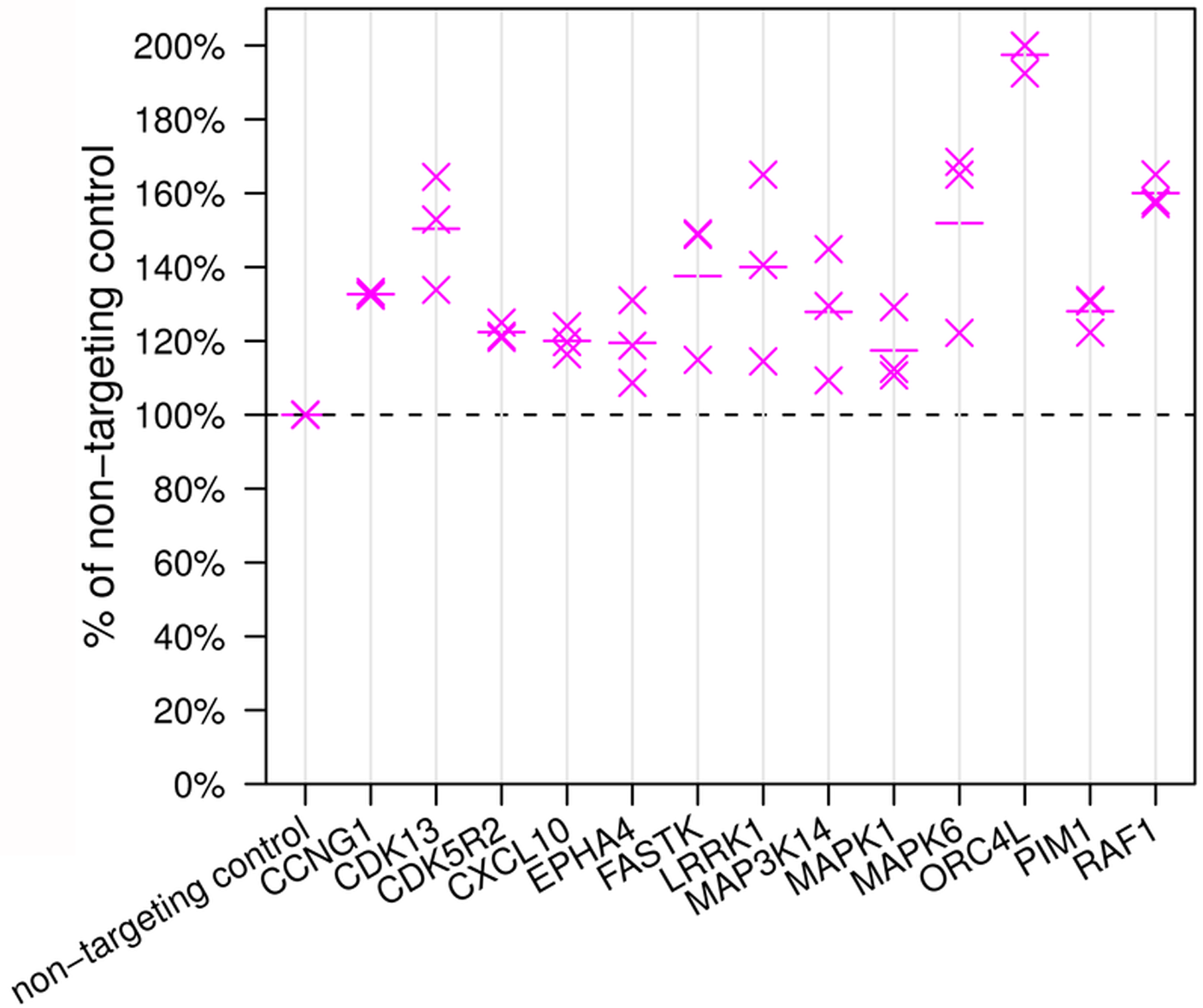

Supplement: Figure S5 — Marginally significant activators (significance level p<0.10 in one-sided t-test) identified in the CTB screening. Crosses indicate the values of siRNA transfected samples as the percentage of negative controls in three biological replicates, and the short horizontal bars indicate the mean value of the replicates. (PNG) [file pone.0022176.s005.png]

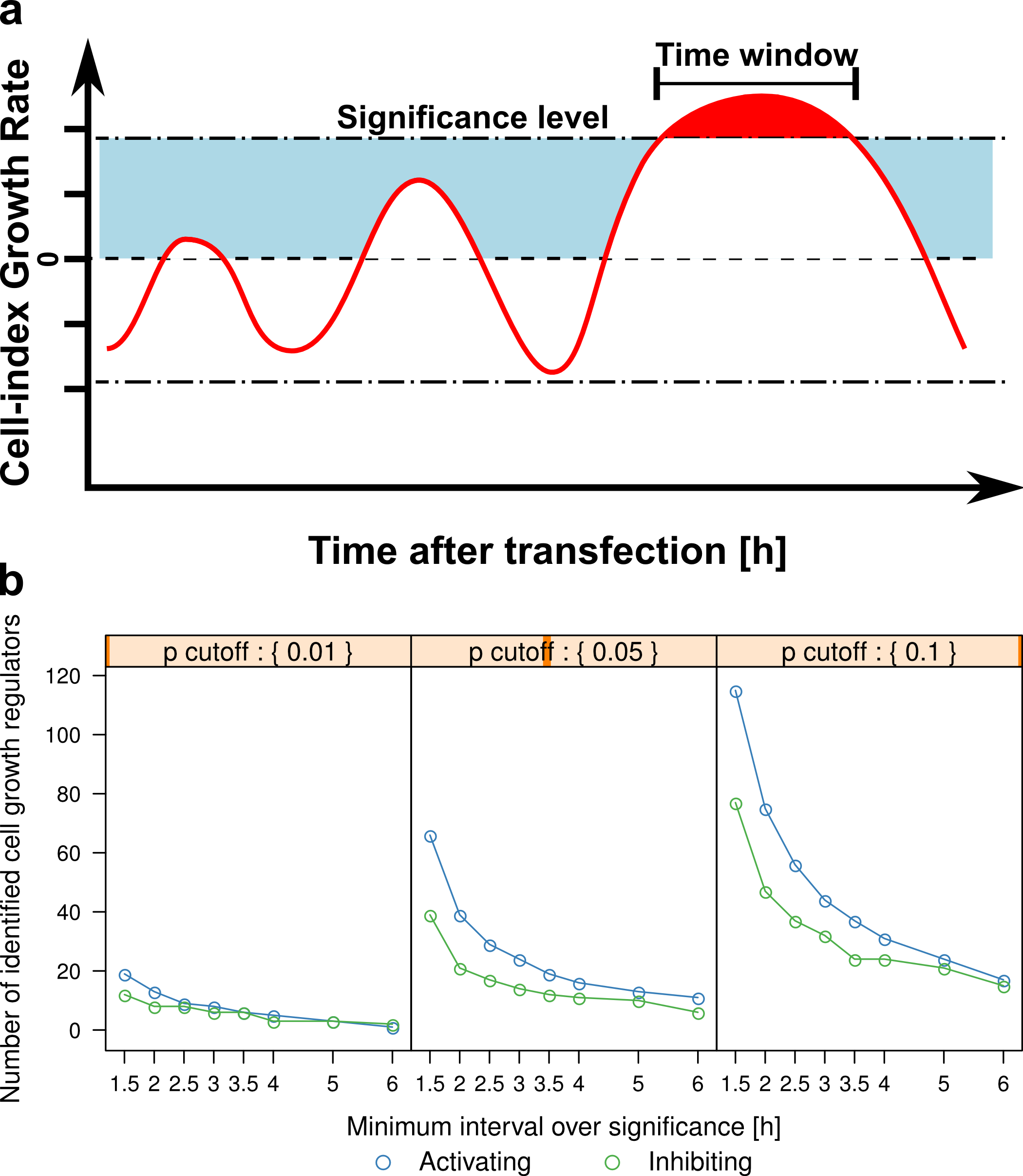

Supplement: Figure S6 — The principle of the flooding watershed algorithm and the number of hits on different parameter selections. (a) For the sake of simplicity, we only discuss selecting the positive regulators, the sample principle however also applies to negative hits. To select transient cell-growth modulators, one puts water sources in each regional minimum (blue regions) of the curves in positive, and flood the relief from the sources. To determine whether a cell-index growth rate curve has a transient significant region, two parameters have to be defined: the significance level (analogously the water level) and the minimum time window in which the CIGR is always over the significance level, both shown in the figure. Using a time-window instead of a single time point helps to eliminate too transient hits (for example those ones that are significant at only one of the 91 measurement points). It is obvious that the higher the significance level is, or the wider the time window is, the less hits will be identified. (b) The number of identified cell growth regulators (both activating and inhibiting) when adjusting the two parameters of the algorithm introduced in (a). The time-window has been set from 1.5 hours (3 measurement points) to 6 hours (12 measurement points), and the p value cut-off has been set to 0.01, 0.05 and 0.10 (corresponding to the z-score cut-off of |z|>1.64, |z|>1.96 and |z|>2.33 respectively). As expected, the number of hits reduce as the p-cutoff becomes more strict as well as the minimum time interval over significance becomes longer. (PNG) [file pone.0022176.s006.png]

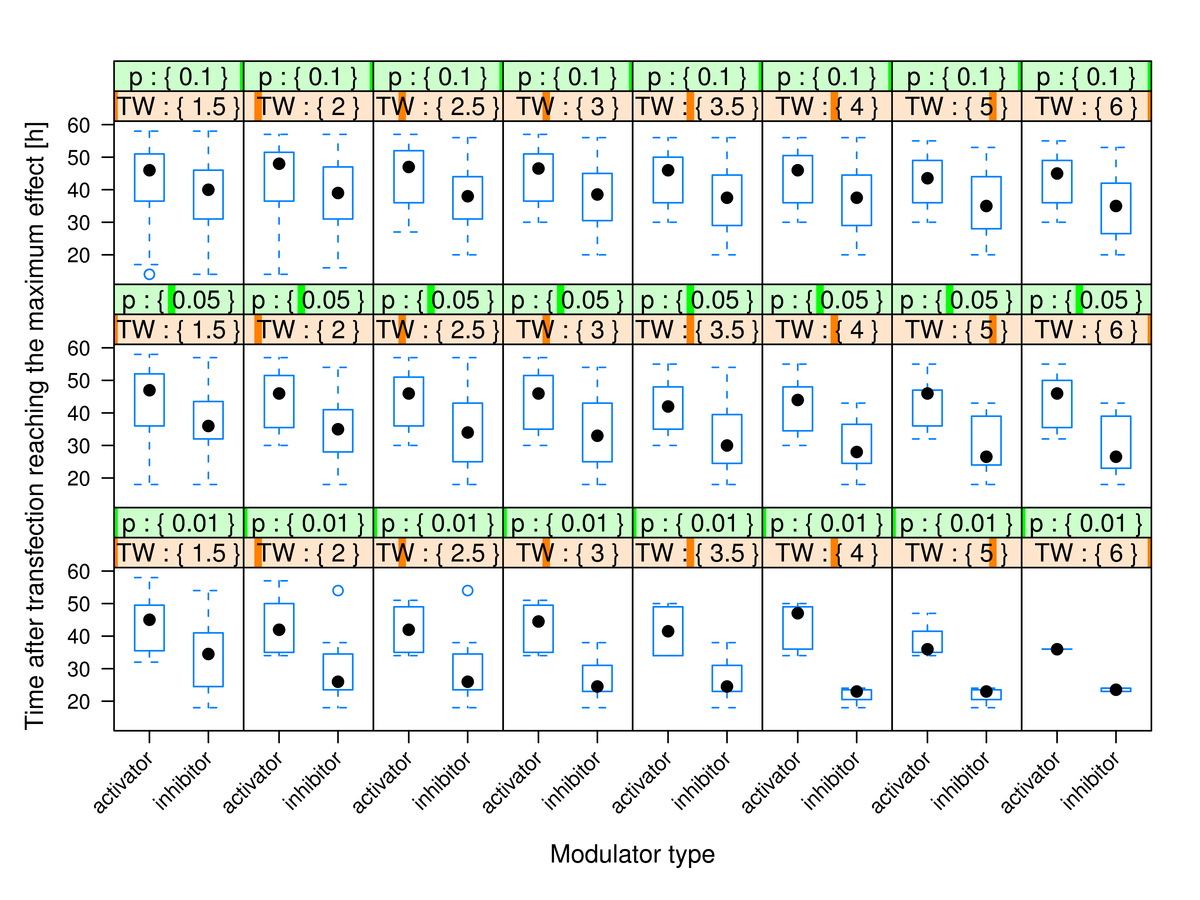

Supplement: Figure S7 — The average time to reach the maximum effect on the cell-index growth rate of inhibitors is shorter than that of activators, independent of the parameter selection. Similar as in the Figure S6, we tested 24 combinations of the significance level (p<0.01, p<0.05 and p<0.10) and the time-window interval (shortened as TW, 1.5 hour to 6 hours), and compared the distribution of the time after transfection at which the siRNA knockdown reaches the maximum effect on the CIGR. In all the cases we observe the average time required by the inhibitors is shorter than the activators (p<0.05 for all the combinations, Student' t-test). (PNG) [file pone.0022176.s007.png]

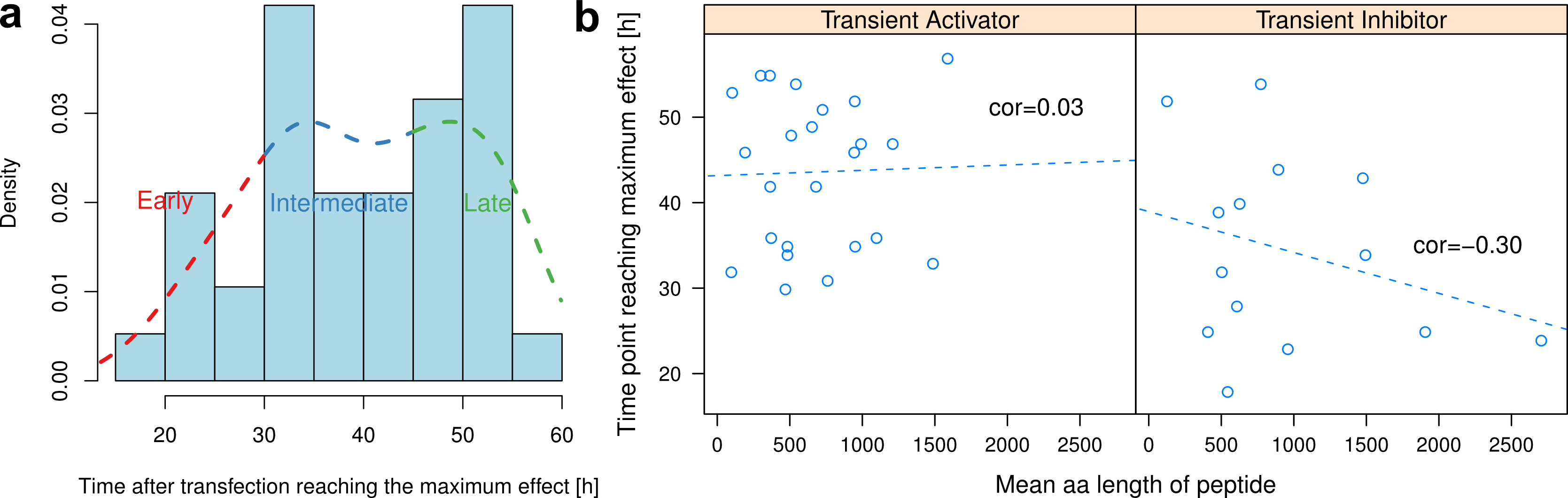

Supplement: Figure S8 — Distribution of time after transfection at which the siRNA knock-down reaches the maximum effect on the cell-index growth rate (‘peak-time’) is linked to the biological function but not the protein size. (a) The distribution of the ‘peak-time’ of all the significant (p<0.05, time-window >3 h) transient modulators of cell growth, which can be divided into three groups: early (<30 hour), intermediate (>30 and <45 hour) and later effectors (>45 hour). The early phase differ from the other two phases in the way that it includes the inhibitory effectors exclusively. (b) The time at which the siRNA knockdown reaching the maximum effect is not significantly linearly correlated with the protein size, neither for the activators or the inhibitors. However, as suggested by the function enrichment analysis and the network analysis of hits discussed in the main text, the peak-time is indeed correlated with the biological function of the genes. (PNG) [file pone.0022176.s008.png]

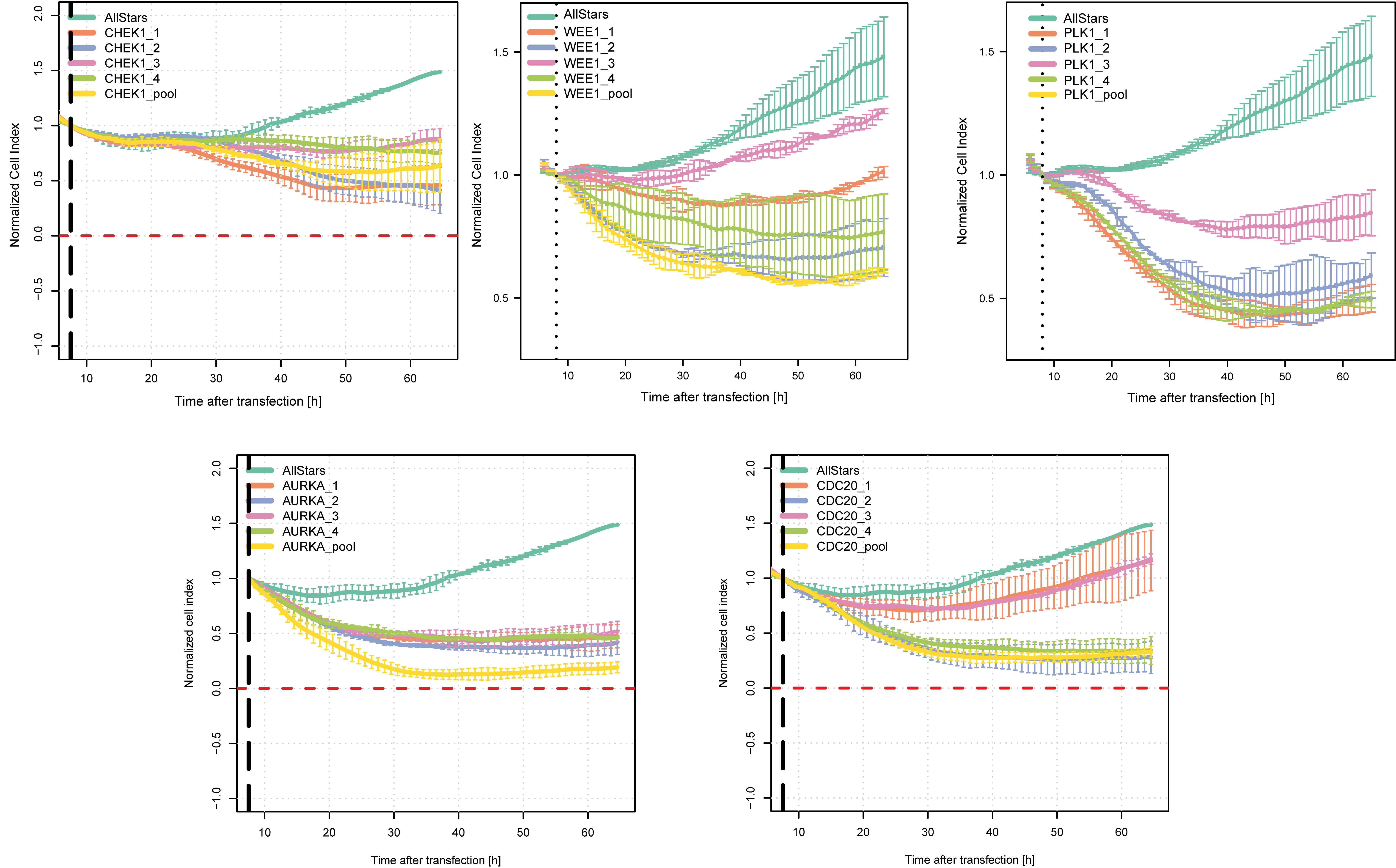

Supplement: Figure S9 — Normalized cell index for five genes identified in the primary RTCA screen as inhibitors of cell proliferation (Figure 4). Pools of four individual siRNAs had been transfected in the primary screen and were deconvoluted to also test the siRNAs (indicated with _1 to _4) individually. AllStars negative control was included in all experiments as negative control. Dotted vertical lines at the 8 hour timepoint indicate the time used for normalization of data. (PNG) [file pone.0022176.s009.png]

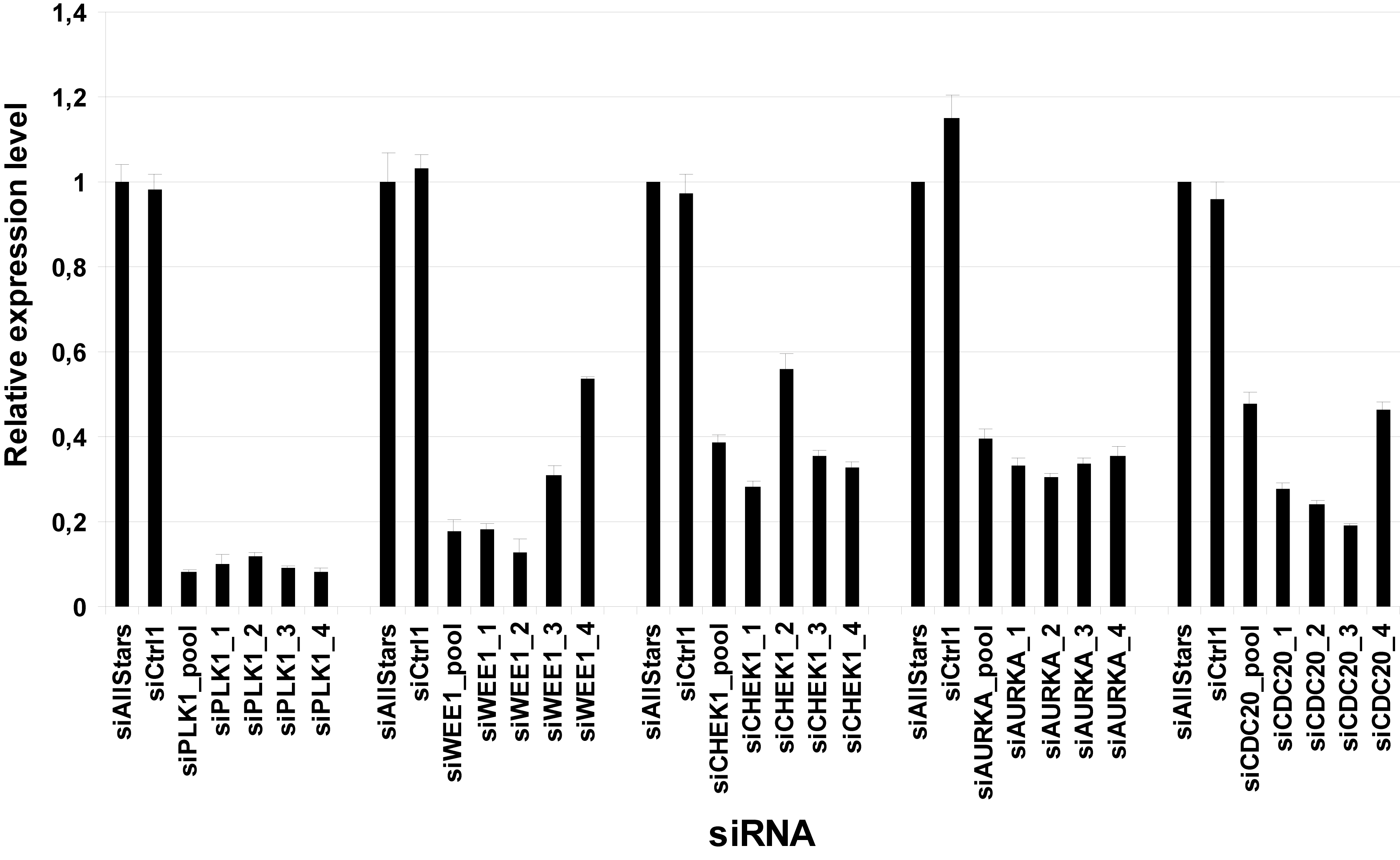

Supplement: Figure S10 — Efficiency of mRNA knockdown of RTCA inhibitor hits measured by qRT-PCR. Pools of four individual siRNAs had been transfected in the primary screen and were deconvoluted to also test the siRNAs (indicated with _1 to _4) individually. SiAllStars negative control as well as siGenome non-targeting control pool #1 were included in all experiments as negative controls. Data was normalized to the effects induced by AllStars control. Results of three technical replicates are shown and error bars indicate the standard deviation. (PNG) [file pone.0022176.s010.png]

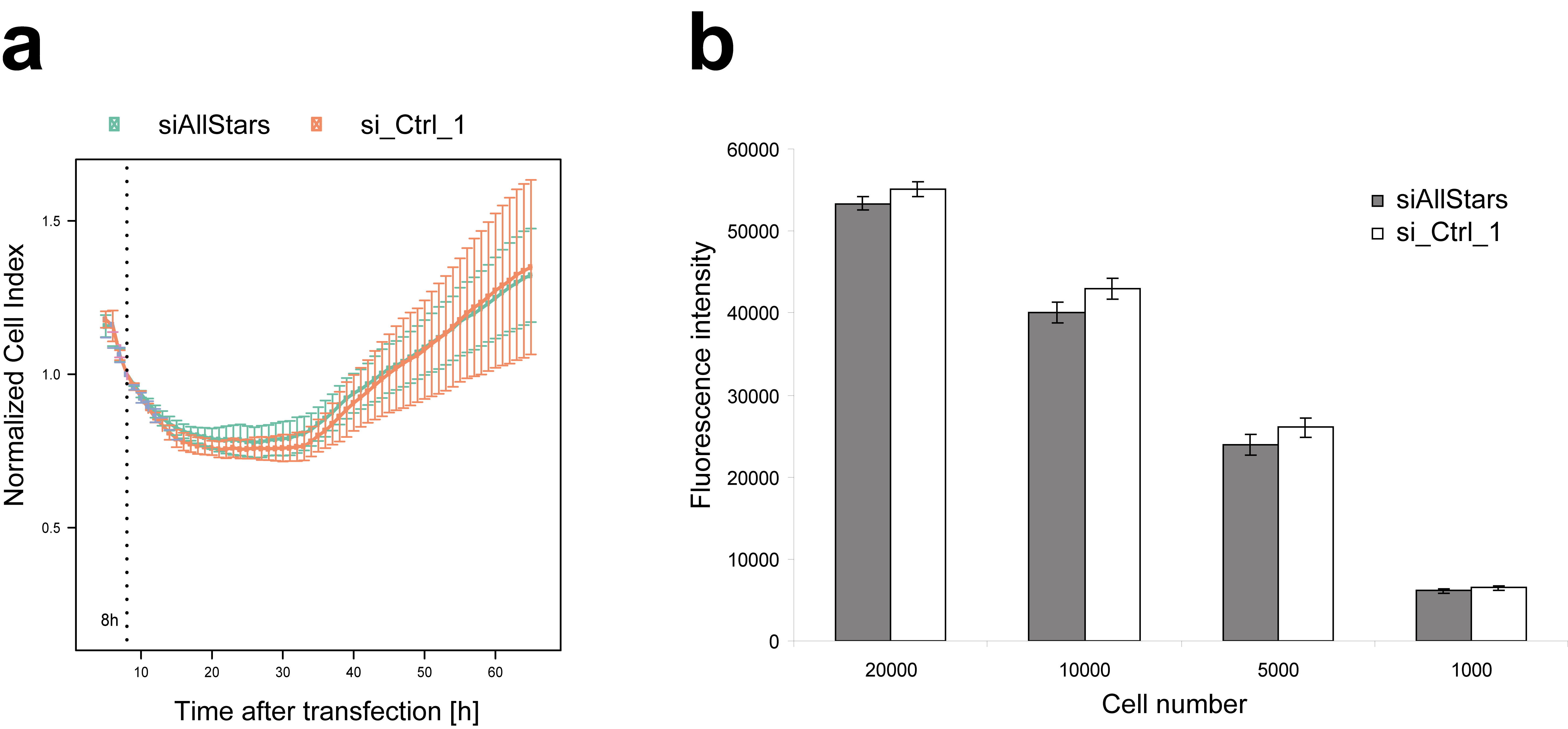

Supplement: Figure S11 — Comparison of negative control siRNAs. SiAllStars and siGenome non-targeting control pool #1 siRNAs were transfected into HeLa cells, and induced effects were monitored by RTCA (a) and CTB (b) assays. Results of six biological replicates are shown and error bars indicate the standard deviation. In the CTB assay the indicated numbers of cells were transfected, 10,000 cells were seeded in the RTCA assay. (PNG) [file pone.0022176.s011.png]
